# WASP integrates substrate topology and cell polarity to guide neutrophil migration

**DOI:** 10.1083/jcb.202104046

**Published:** 2021-12-29

**Authors:** Rachel M. Brunetti, Gabriele Kockelkoren, Preethi Raghavan, George R.R. Bell, Derek Britain, Natasha Puri, Sean R. Collins, Manuel D. Leonetti, Dimitrios Stamou, Orion D. Weiner

**Affiliations:** 1 Cardiovascular Research Institute, University of California, San Francisco, San Francisco, CA; 2 Department of Biochemistry and Biophysics, University of California, San Francisco, San Francisco, CA; 3 Center for Geometrically Engineered Cellular Membranes, University of California, San Francisco, San Francisco, CA; 4 Department of Chemistry, University of Copenhagen, Copenhagen, Denmark; 5 Center for Geometrically Engineered Cellular Membranes, University of Copenhagen, Copenhagen, Denmark; 6 University of California, Berkeley–University of California, San Francisco Graduate Program in Bioengineering, University of California, San Francisco, San Francisco, CA; 7 Chan Zuckerberg Biohub, San Francisco, CA; 8 Department of Microbiology and Molecular Genetics, University of California, Davis, Davis, CA

## Abstract

To control their movement, cells need to coordinate actin assembly with the geometric features of their substrate. Here, we uncover a role for the actin regulator WASP in the 3D migration of neutrophils. We show that WASP responds to substrate topology by enriching to sites of inward, substrate-induced membrane deformation. Superresolution imaging reveals that WASP preferentially enriches to the necks of these substrate-induced invaginations, a distribution that could support substrate pinching. WASP facilitates recruitment of the Arp2/3 complex to these sites, stimulating local actin assembly that couples substrate features with the cytoskeleton. Surprisingly, WASP only enriches to membrane deformations in the front half of the cell, within a permissive zone set by WASP’s front-biased regulator Cdc42. While WASP KO cells exhibit relatively normal migration on flat substrates, they are defective at topology-directed migration. Our data suggest that WASP integrates substrate topology with cell polarity by selectively polymerizing actin around substrate-induced membrane deformations in the front half of the cell.

## Teaser

Brunetti et al. demonstrate how physical and biochemical signals cooperate to pattern actin polymerization in motile neutrophils. Membrane deformations in the front half of the cell recruit WASP and elicit actin polymerization, linking substrate features with the cytoskeleton. These properties reveal a role for WASP in substrate-guided migration.

## Introduction

Motile cells must coordinate many types of actin networks to achieve directed migration (Blanchoin et al., 2014). Nucleation promoting factors (NPFs) control when and where branched actin is assembled, with different NPFs giving rise to different types of actin networks (Pollitt and Insall, 2009; Rottner et al., 2017). For example, Wiskott–Aldrich syndrome protein (WASP) family verprolin-homologous protein (WAVE) forms broad, propagating waves at the leading edge that pattern the sheet-like actin networks that generate lamellipodia (Weiner et al., 2007; Veltman et al., 2012; Leithner et al., 2016). In contrast, neural WASP (N-WASP) has a punctate distribution and forms finger-like actin networks that comprise podosomes (Isaac et al., 2010; Nusblat et al., 2011; Mizutani et al., 2002), invadopodia (Yamaguchi et al., 2005; Yu et al., 2012; Yu and Machesky, 2012), and sites of endocytosis (Kessels and Qualmann, 2002; Merrifield et al., 2004; Benesch et al., 2005). While the function of WAVE and N-WASP is well understood, other NPFs remain poorly characterized. This includes WASP, an N-WASP homologue specific to the hematopoietic lineage. WASP was initially identified through its role in Wiskott–Aldrich syndrome (Derry et al., 1994), an immune disorder characterized by abnormal platelets, eczema, and recurrent infection (Wiskott, 1937; Aldrich et al., 1954). However, despite the homing defects of immune cells in WASP-deficient patients and animal models (Ochs et al., 1980; Snapper et al., 2005; de Noronha et al., 2005; Westerberg et al., 2005; Jones et al., 2013), how WASP contributes to actin organization and cell migration remains poorly understood.

The function of WASP in vertebrates has largely been inferred from the function of its homologue N-WASP. However, important functional divergences have been reported between these proteins (Isaac et al., 2010; Nusblat et al., 2011). In particular, N-WASP cannot compensate for the loss of WASP in T cell chemotaxis (Jain and Thanabalu, 2015). The basis of WASP’s role in immune cell guidance remains unknown. Do functions ascribed to N-WASP, such as endocytosis and podosome/invadopodia formation, also underlie WASP-deficient immune cell migration defects, or does WASP participate in additional aspects of physiology during immune cell migration? The functional roles of actin nucleators and NPFs have successfully been elucidated through analysis of spatiotemporal dynamics and loss-of-function phenotypes (Bear et al., 1998; Rogers et al., 2003; Kunda et al., 2003; Weiner et al., 2007; Miki et al., 1998; Nakagawa et al., 2001; Taunton et al., 2000; Benesch et al., 2002; Duleh and Welch, 2010; Zuchero et al., 2009; Sagot et al., 2002; Yang et al., 2007; Manor et al., 2015). Here, we use this approach to probe WASP’s contribution to neutrophil migration.

## Results

### WASP enriches to puncta that associate with the substrate

**Figure S1.**
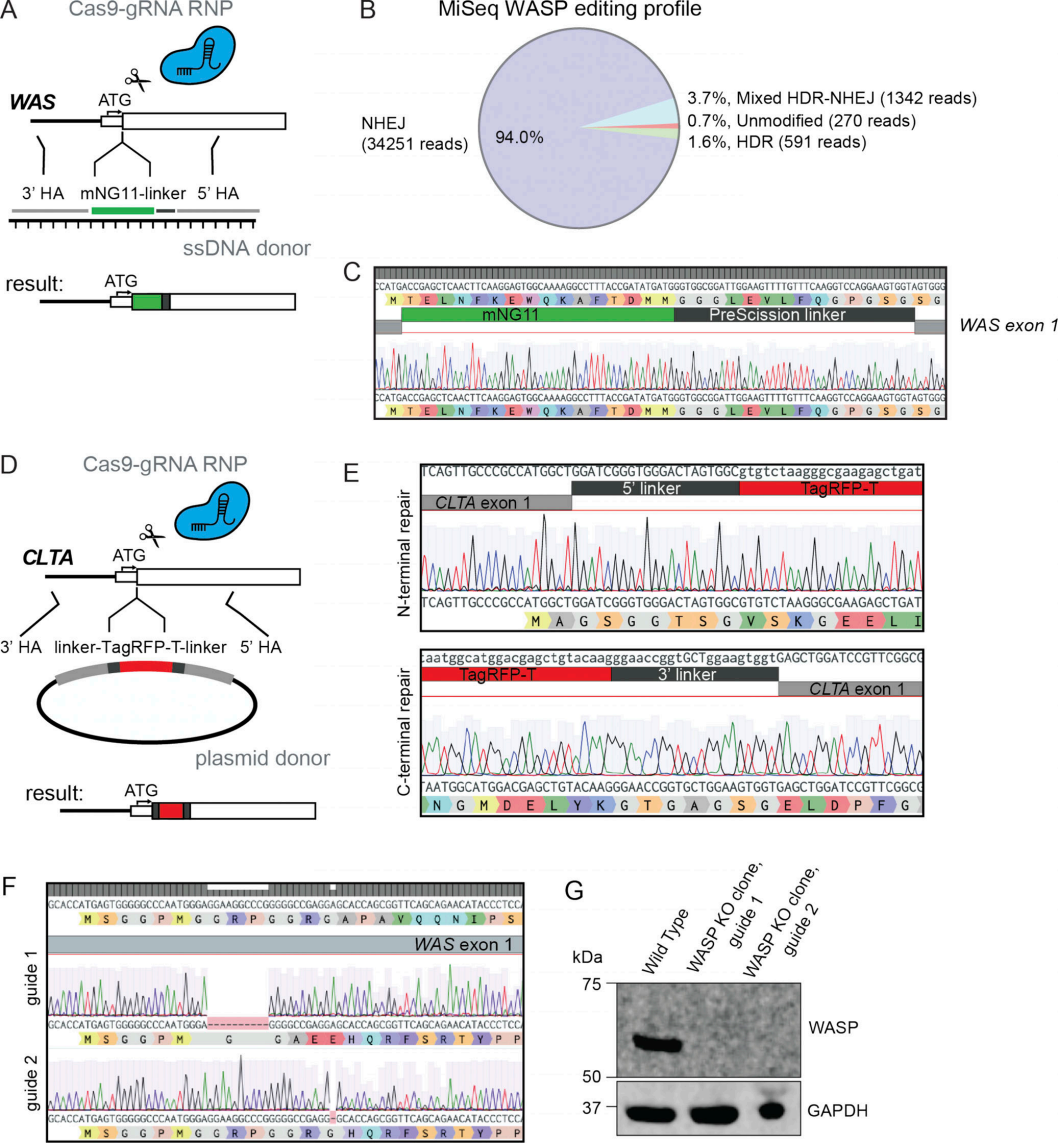
**Genetic engineering of HL-60 cells.** **(A)** Strategy for endogenous tagging of WASP with split mNG2 using a Cas9–sgRNA RNP complex and ssDNA donor. **(B)** MiSeq results from A reveal high cutting efficiency in HL-60s but poor homology-directed repair (HDR). Analysis of paired end reads was done with CRISPResso2 (Clement et al., 2019). **(C)** Sequencing of WASP KI cells confirms correct insertion of mNG2_11_ tag. **(D)** Strategy for endogenous tagging of clathrin LCa with full-length TagRFP-T using a Cas9–sgRNA RNP complex and plasmid donor. **(E)** Sequencing of an isolated homozygous *CLTA* KI clone shows correct repair at both ends of the cut site. **(F)** Sequence validation of the two clonal WASP KO lines assayed. Both lines have deletions that lead to a frame shift, nonsense, and termination following the end of exon 1. **(G)** Western blot confirms that WASP is absent in both assayed clonal lines. NHEJ, nonhomologous end joining.

Because of its key role in the immunological disorder Wiskott–Aldrich syndrome, WASP has traditionally been studied through its loss-of-function phenotypes in immune cells from patients and animal models of the disease (Ochs et al., 1980; Snapper et al., 2005; de Noronha et al., 2005; Westerberg et al., 2005; Jones et al., 2013). Less attention has been paid to its localization and dynamics in cells. Therefore, we turned to the easily manipulated human neutrophil–like cell line HL-60 to better understand the spatiotemporal dynamics of WASP and how these attributes might inform its function. In a previous report, exogenously expressed WASP was shown to enrich to the tip of the leading edge and to membrane-associated puncta in HL-60s (Fritz-Laylin et al., 2017). However, protein overexpression can lead to ectopic localization (Doyon et al., 2011). To better profile WASP’s functional properties, we generated a fluorescently tagged protein at the endogenous locus in HL-60 cells using CRISPR-Cas9 and homology-directed repair (Fig. S1, A–C).

**Figure 1.**
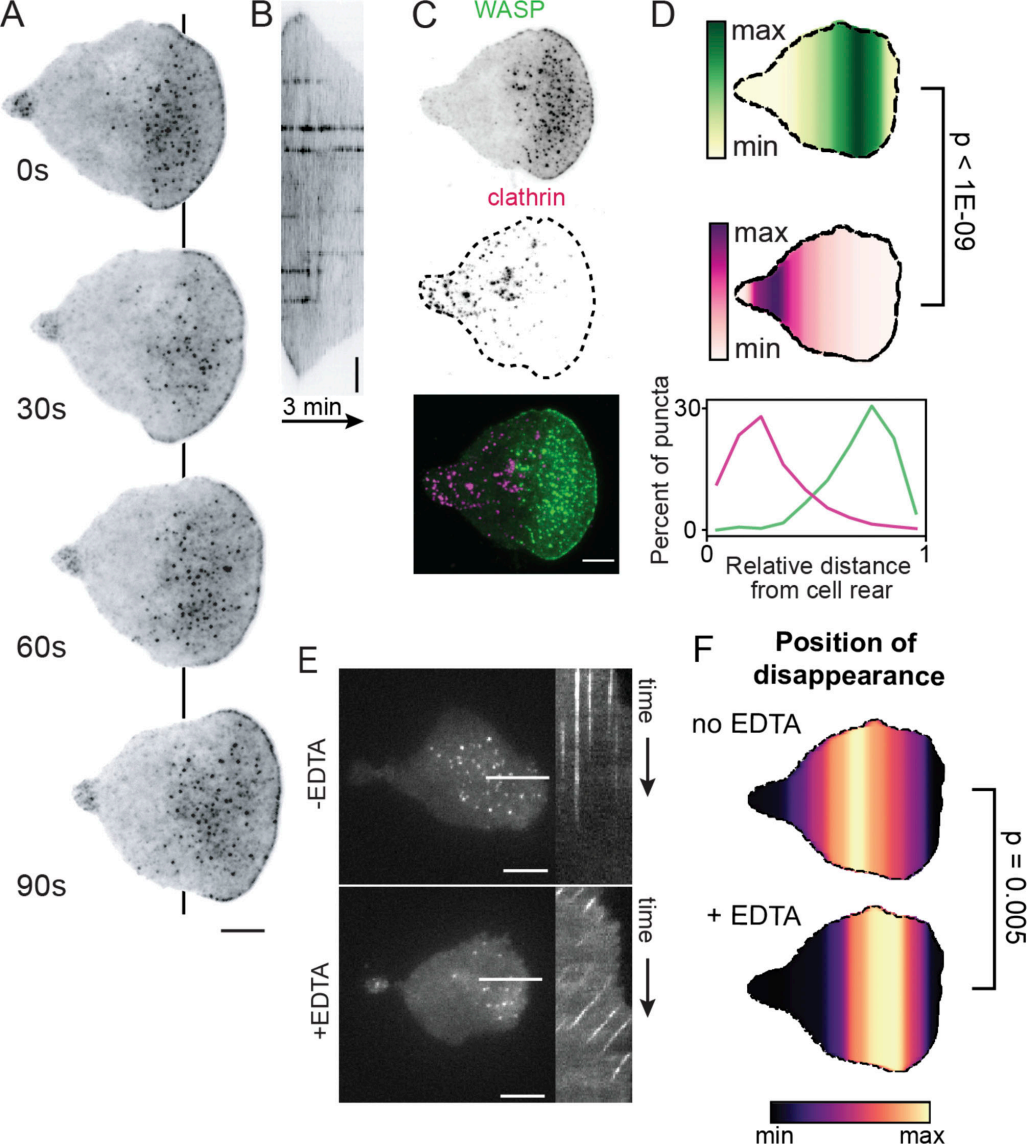
**Endogenous WASP forms puncta stabilized by cell-substrate interactions.** **(A)** TIRF microscopy of endogenous WASP reveals localization to the tip of the lamellipod and to ventral puncta throughout the front half of the cell and to a lesser extent the uropod. Color map is inverted. Each frame is 30 s. See also Video 1. **(B)** Kymograph along the line in A highlights the stationary nature of WASP puncta relative to the substrate. Scale bar is 2 μm. **(C)** Endogenous WASP and endogenous clathrin LCa fail to colocalize. Color map is inverted for single-channel images. See also Video 2. **(D)** Spatial distribution of WASP (green) and clathrin (magenta) puncta and their corresponding line scans confirm consistent spatial separation of these proteins in coexpressing cells. *n* = 11 cells collected across three experiments with >1,000 puncta measured for each marker. Mean relative position of WASP is 0.71 ± 0.01 and mean relative position of clathrin is 0.29 ± 0.02 on the single-cell level. P = 8.82E-10 by a paired two-tailed *t* test on the mean puncta position of each marker in each cell. **(E)** Treatment of cells with EDTA, a calcium and magnesium chelator that blocks integrin-based adhesion, causes normally stationary WASP puncta to experience retrograde flow relative to the substrate. Left depicts a single frame of endogenous WASP with a line used to construct the kymograph to the right. See also Video 3. **(F)** Spatial distribution of the position of disappearance of WASP puncta reveals earlier extinction in the presence of EDTA. *n* = 12 cells per condition collected across three experiments with ∼300 puncta measured for each condition. Mean relative position of puncta disappearance is 0.55 ± 0.03 in the absence of EDTA and 0.66 ± 0.02 in the presence of EDTA on the single-cell level. P = 0.005 by an unpaired two-tailed *t* test on the mean position of puncta disappearance for each cell. For relative positions, the cell rear is defined as 0 and the cell front as 1. Scale bars are 5 μm unless otherwise specified. max, maximum; min, minimum.

**Figure S2.**
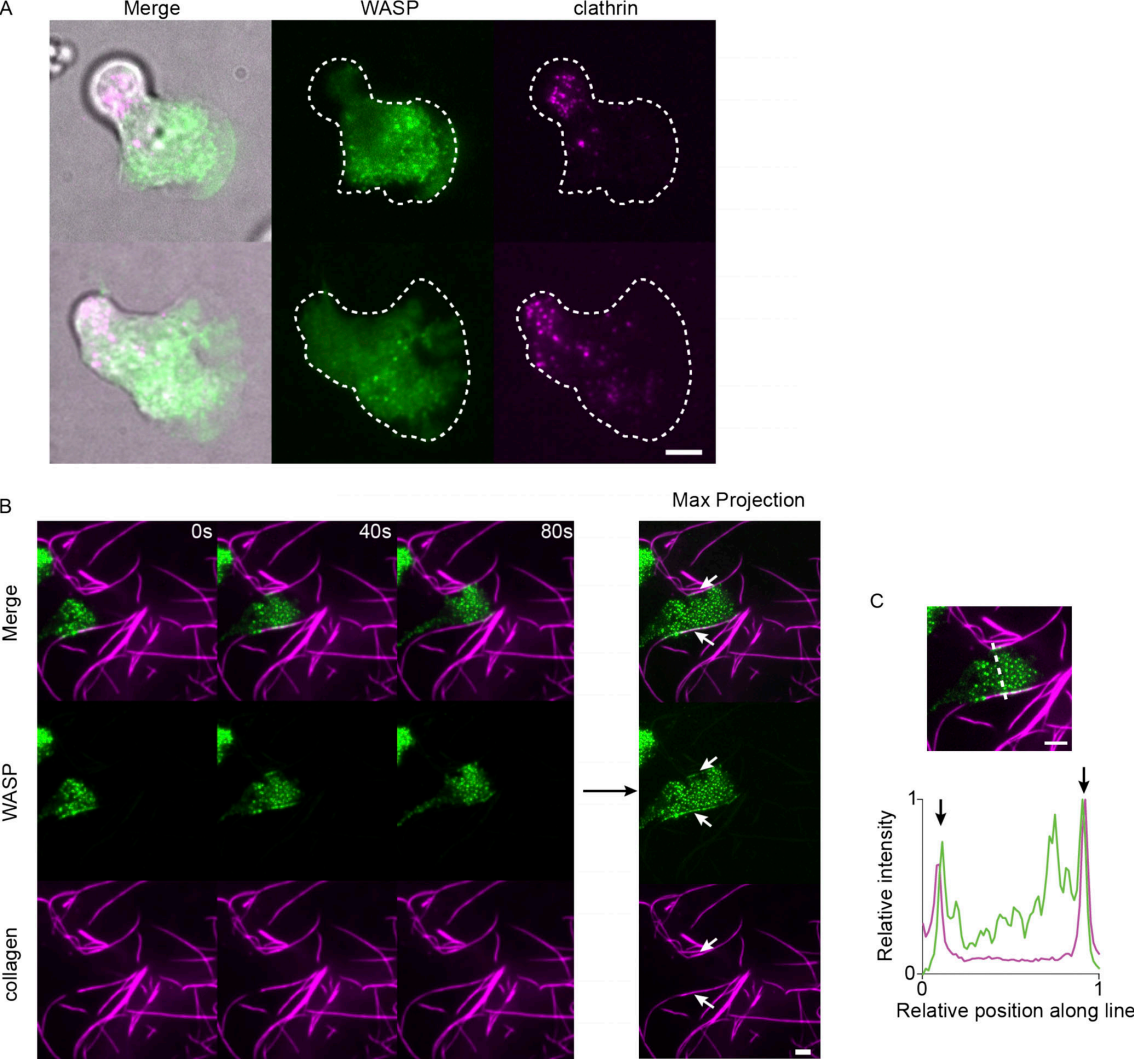
**WASP puncta formation in the absence of agarose overlay and on collagen fibers.** **(A)** Endogenous WASP forms front-biased puncta and is spatially segregated from clathrin LCa in the absence of agarose overlay. **(B)** As a more physiological counterpart to bead-induced membrane invaginations, we imaged WASP in cells migrating across fluorescent collagen-coated coverslips and found that WASP is recruited to sites of cell contact with fibers. Right-hand side shows the maximum (max) intensity projection over time, which further highlights the enrichment of WASP to cell–fiber interfaces. Stretches of contact are marked with arrows. **(C)** Relative background-subtracted WASP (green) and collagen (magenta) signal along the dashed line drawn on the top image. Arrows denote WASP puncta formed at sites of cell–collagen contact. Scale bars are 5 μm. Imaged with TIRF microscopy.

We used an under-agarose preparation to visualize the ventral surface of WASP knockin (KI) HL-60 cells with total internal reflection fluorescence (TIRF) microscopy. Endogenous WASP localized to membrane-associated puncta and the periphery of the leading edge, a distribution that was broadly consistent with exogenous WASP (Fritz-Laylin et al., 2017). However, enrichment of WASP to puncta in the front half of the cell, separate from the cell periphery, was more strongly pronounced than previously appreciated and proved to be the dominant pattern of WASP localization (Fig. 1 A and Video 1). Additionally, WASP puncta were remarkably stationary, often persisting at the same position relative to the coverslip on the minute time scale, despite bulk cell displacement (Fig. 1 B). Because N-WASP has been implicated in clathrin-mediated endocytosis (CME; Kessels and Qualmann, 2002; Merrifield et al., 2004; Benesch et al., 2005), we investigated whether WASP puncta represent sites of CME. Endogenous WASP and endogenous clathrin light chain A (LCa) failed to colocalize in the front half of the cell (Fig. 1, C and D; Fig. S1, D and E; Fig. S2 A; and Video 2). In addition, both clathrin-dependent and -independent endocytic pathways have been shown to occur at the rear of polarized HL-60 cells (Davis et al., 1982; Subramanian et al., 2018). Therefore, front-biased WASP puncta do not appear to mark sites of endocytosis.

**Video 1.** **Distribution of endogenous WASP-mNeonGreen2 in a persistently migrating HL-60 cell (as in ****Fig. 1 A****).** WASP enriches to foci on the ventral surface of the cell that are concentrated toward the cell front. Imaged with TIRF microscopy and displayed with an inverted color map. Scale bar is 5 μm. Time, min:s. Video is displayed at 10 frames per second (fps).

**Video 2.** **Endogenous WASP-mNeonGreen2 and endogenous clathrin LCa–TagRFP-T remain spatially separate in a migrating HL-60 cell (as in ****Fig. 1 D****).** Scale bar is 5 μm. Imaged with TIRF microscopy. Time, min:s. Video is displayed at 2 fps.

Other documented roles for WASP/N-WASP include the formation of invasive structures (Yu et al., 2012; Yu and Machesky, 2012; Isaac et al., 2010; Nusblat et al., 2011; Mizutani et al., 2002; Yamaguchi et al., 2005) and the facilitation of cell adhesion (Misra et al., 2007; Zhang et al., 2006) and spreading (Misra et al., 2007; Liu et al., 2013). Neutrophils have a limited capacity for generating invadopodia or podosomes, failing to invade soft substrates like Matrigel or to form their characteristic actin–vinculin rosettes (Cougoule et al., 2012). We therefore investigated whether WASP puncta are linked to cell adhesion. While neutrophils do not generate the long-lived focal adhesions found in many adherent cell lines (Yürüker and Niggli, 1992; Lämmermann and Sixt, 2009), we hypothesized that the fixed location of WASP puncta relate to adhesion with the underlying substrate. To test this, we imaged cells in the presence of EDTA, a calcium and magnesium chelator that blocks integrin–ligand binding and therefore prevents adhesion (Zhang and Chen, 2012). In thin slide preparations where cells can touch both slide and coverslip, leukocytes can migrate in an adhesion-independent manner (Malawista et al., 2000). In the absence of integrin-based adhesion, we found that WASP puncta continued to form on the cell surface but were no longer stationary relative to the substrate and instead began undergoing retrograde flow (Fig. 1 E; and Video 3). Additionally, WASP puncta are less stable for cells lacking integrin adhesions, extinguishing significantly closer to their point of nucleation at the cell front (Fig. 1 F). The induced motility of WASP puncta when cell–substrate adhesions are removed suggests that stationary WASP puncta normally depend on engagement with the substrate. This finding is supported by previous observations that WASP knockout (KO) neutrophils have an impaired ability to adhere to and cross cell monolayers under shear flow (Zhang et al., 2006), while neutrophils expressing only constitutively active WASP demonstrate increased adhesion and migration in flow chambers (Keszei et al., 2018). We next asked which substrate features are involved in WASP recruitment.

**Video 3.** **WASP forms puncta that are stationary relative to the substrate but undergo retrograde flux in the absence of integrin engagement (+EDTA; as in ****Fig. 1 E****).** Scale bar is 5 μm. Imaged with TIRF microscopy. Video is displayed at 30 fps.

### WASP is curvature sensitive and sorts to sites of saddle curvature

**Figure 2.**
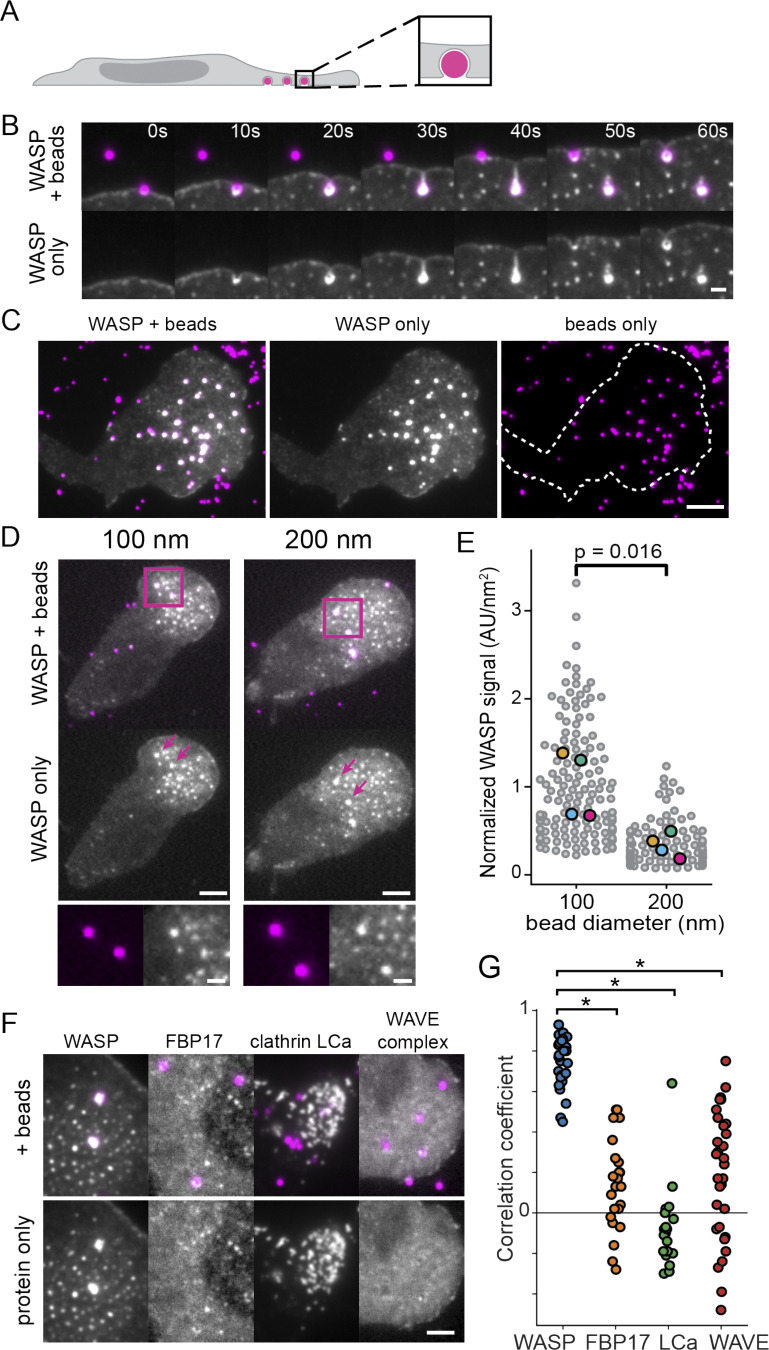
**WASP is recruited to membrane invaginations in a curvature-dependent manner.** **(A)** Experimental strategy of plating cells on beads to induce sites of substrate-controlled plasma membrane curvature. **(B)** Time-lapse imaging reveals rapid recruitment of endogenous WASP to 500-nm bead-induced invaginations. Scale bar is 1 μm. **(C)** WASP consistently localizes to 200-nm bead-induced invaginations across the front half of the cell. Scale bar is 5 μm. See also Video 4. **(D)** WASP KI cells were plated on beads of varying diameter to assess the curvature sensitivity of WASP recruitment. Images are scaled to the same intensity for WASP. Below is a zoomed inset of the boxed regions. Scale bars are 5 μm and 1 μm for the inset. **(E)** Quantification of WASP signal at 100- and 200-nm beads reveals significantly more WASP at smaller invaginations (sites of higher curvature) when normalized to bead surface area. Mean WASP signal per unit area is 1.01 ± 0.19 a.u./nm^2^ for 100-nm beads and 0.34 ± 0.07 a.u./nm^2^ for 200-nm beads on the replicate level. *n*_100 nm_ = 142 beads and *n*_200 nm =_ 120 beads each collected across four experiments. Large markers denote the mean of each replicate, which were compared using an unpaired two-tailed *t* test (P = 0.016). **(F)** WASP enrichment to 500-nm beads is much stronger than that of other known curvature-sensitive proteins FBP17, clathrin LCa, and WAVE complex. Scale bar is 2 μm. **(G)** Pearson correlation coefficients for 1.5 × 1.5-μm regions of interest of a bead and the protein of interest. WASP has a significantly higher correlation with bead signal (*r* = 0.75 ± 0.02) compared with FBP17 (0.14 ± 0.05), clathrin LCa (−0.09 ± 0.04), and WAVE complex (0.19 ± 0.06). The correlation of each protein with beads was compared with the correlation of WASP with beads using an unpaired two-tailed *t* test. Asterisks mark significance. P_WASP/FBP17_ = 6.56E-18, P_WASP/clathrin_ = 4.60E-24, and P_WASP/WAVE_ = 5.58E-13. *n*_WASP_ = 31, *n*_FBP17_ = 24, *n*_clathrin_ = 21, and *n*_WAVE_ = 31 beads each from one experiment. AU, arbitrary units.

Positive (inward) membrane curvature can organize N-WASP both in reconstituted systems (Takano et al., 2008) and in live cells plated on nanopatterns (Lou et al., 2019). We sought to test whether inward curvature similarly templates WASP organization. Because WASP/N-WASP natively associates with spherical membrane invaginations like endocytic pits, we used polystyrene beads as our membrane-deforming agents (Fig. 2 A). The use of spherical beads enabled us to explore 2D curvatures that are inaccessible with most fabricated nanopatterns. Specifically, beads allowed us to probe (1) two dimensions of positive (inward) isotropic curvature at the body of the bead-induced invagination and (2) saddle curvature at the invagination neck. Because curvature-sensitive proteins can discriminate between 1D and 2D curvatures (Iversen et al., 2015; Larsen et al., 2020), it is important to have tools that can probe 2D curvature in cells. Additionally, this approach has the benefit of being highly tunable through the availability of beads of different diameters (and therefore radii of curvature). Finally, use of commercially available, inert beads removed the cost and throughput limitations of nanofabrication techniques, like electron beam lithography (Li et al., 2019).

As HL-60s migrated over beads, WASP was recruited within seconds and persisted at the beads while they were under the front half of the cell (Fig. 2, B and C; and Video 4). To determine whether this enrichment is curvature sensitive, we compared WASP recruitment in cells migrating over 100- versus 200-nm-diameter beads (Fig. 2 D). In vitro, enrichment of curvature-sensitive proteins to different-sized liposomes is normalized per unit area (Bhatia et al., 2009). Applying this normalization scheme to our data, we found a threefold enrichment of WASP to the 100-nm beads (higher radius of curvature) compared with the 200-nm beads (Fig. 2 E). The enrichment to higher radii of curvature is consistent with the behavior of curvature-sensitive proteins, including BAR (Bin/amphiphysin/Rvs) domain–containing proteins (Bhatia et al., 2009) and components of CME (Zhao et al., 2017), suggesting that WASP recruitment is sensitive to curvature. To probe whether membrane deformation could similarly be an organizing cue for physiological substrates, we plated cells on coverslips coated with a thin layer of fluorescent collagen fibers (Elkhatib et al., 2017). Cells invaded the matrix and were deformed by the collagen fibers, which elicited endogenous WASP recruitment to cell–fiber interfaces (Fig. S2, B and C).

**Video 4.** **Endogenous WASP (gray) is recruited to all 200-nm beads (magenta) as the cell encounters them (as in ****Fig. 2 C****).** WASP signal is sustained over the front half of the cell but diminishes as beads reach the cell rear. Scale bar is 5 μm. Imaged with TIRF microscopy. Time, min:s. Video is displayed at 7 fps.

**Figure S3.**
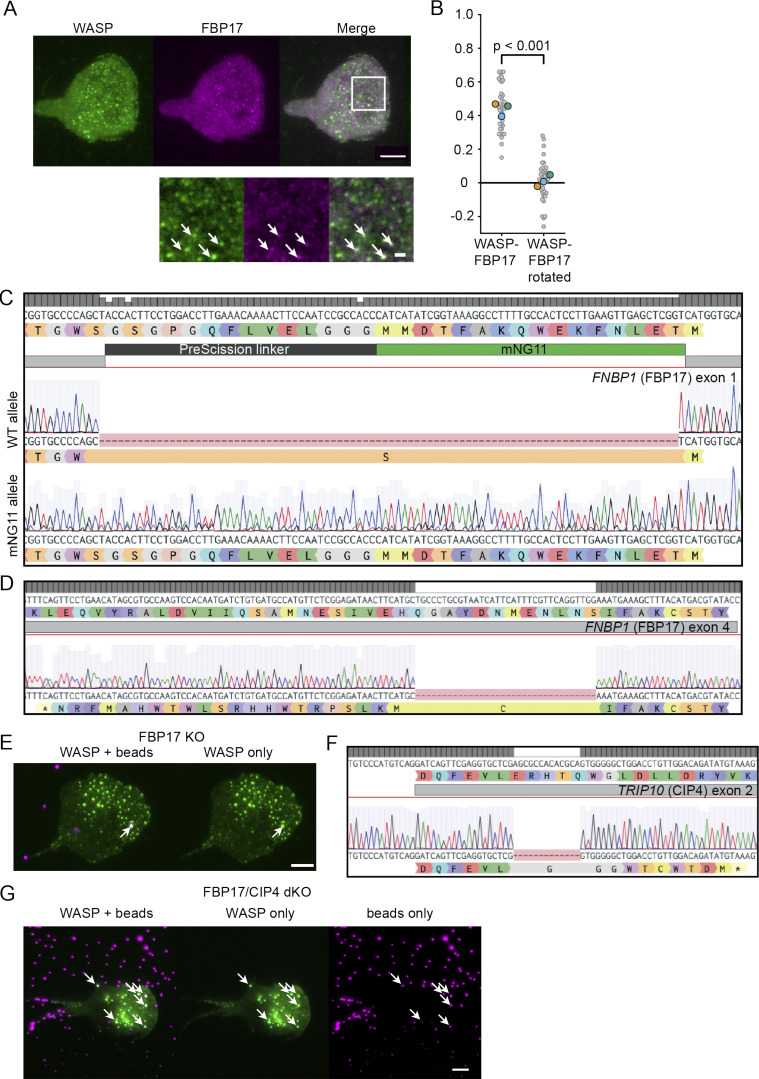
**FBP17 colocalizes with WASP, but neither FBP17 nor CIP4 are required for WASP curvature sensitivity.** **(A)** Exogenously expressed WASP and endogenous FBP17 colocalize. Below shows a zoomed inset with arrows marking example positions of overlap. Scale bar is 1 μm in the inset. **(B)** Pearson correlation coefficients between WASP and FBP17 reveal significant colocalization (*r* = 0.44 ± 0.02) compared with a 90° rotated control (0.01 ± 0.02). *n* = 38 7.5 × 7.5-μm regions of interest collected over three experiments. P = 1.51E-17 by a paired two-tailed *t* test on the full distribution. **(C)** Sequencing shows a WT allele and a correct insertion of mNG2_11_ in the isolated heterozygous FBP17 KI clone. **(D)** Sequencing of an FBP17 KO line shows homozygous deletion of 34 bp after amino acid 74, which causes a frame shift and early termination that disrupts the BAR domain (amino acids 1–264). **(E)** Exogenous WASP continues to form puncta and enrich to bead-induced invaginations in the absence of FBP17. The arrow marks an example site of WASP recruitment to a 500-nm bead. **(F)** Sequencing of a homozygous Δ13 bp CIP4 KO clone generated in the FBP17 KO background shown in D. **(G)** Endogenous WASP continues to form puncta and enrich to 200-nm bead-induced membrane invaginations in the absence of both FBP17 and CIP4. Arrows mark example sites of WASP recruitment to beads. Scale bars are 5 μm. dKO, double knockout.

WASP was strikingly consistent in its recruitment across multiple beads under the same cell front. In adherent cells plated on nanopatterns, curvature-sensitive proteins are normally heterogeneous in their degree of recruitment to adjacent nanobars (Lou et al., 2019; Zhao et al., 2017). This led us to compare the strength of WASP recruitment with that of other known curvature sensors through measuring enrichment to sites of bead-induced membrane deformation (Fig. 2, F and G). First, we investigated the WASP/N-WASP–interacting, BAR domain-containing protein FBP17. FBP17 activates N-WASP in vitro (Takano et al., 2008) and has been posited as an organizer of N-WASP–dependent actin nucleation in endocytosis (Tsujita et al., 2006) and at the leading edge of polarized cells (Tsujita et al., 2015). In COS-1 cells, FBP17 also exists as membrane-associated puncta that are reminiscent of our WASP puncta (Tsujita et al., 2015). While FBP17 is recruited to sites of induced membrane curvature in U2OS cells plated on nanopillars (Lou et al., 2019), it was only weakly recruited to 500-nm bead-induced invaginations (Fig. 2 G). Additionally, despite colocalization of endogenous FBP17 and WASP at puncta biased toward the cell front (Fig. S3, A–C), FBP17 was required neither for WASP puncta formation nor for WASP recruitment to bead-induced membrane deformations, since these behaviors persisted in FBP17 KO cells (Fig. S3, D and E). FBP17 is part of the TOCA subfamily of BAR domain proteins (composed of FBP17, CIP4, and TOCA-1), which have been shown to co-oligomerize and compensate for one another in other cellular systems (Chan Wah Hak et al., 2018; Tsujita et al., 2013). To assess whether the lack of a phenotype for WASP localization in FBP17 KO cells was due to redundancy within this family, we also knocked out the next highest expressed member of this family, CIP4. Endogenous WASP puncta still continued to form and were triggered by membrane curvature in FBP17/CIP4 double-KO cells (Fig. S3, F and G). We have not yet generated the FBP17/CIP4/TOCA-1 triple KO to investigate whether TOCA-1 (the remaining and lowest-expressed member of this family on the transcriptional level [Rincón et al., 2018]) contributes to WASP recruitment in neutrophils.

We also investigated the effect of bead-induced membrane deformation on the recruitment of other proteins that are known to sense membrane curvature–clathrin LCa (Elkhatib et al., 2017; Zhao et al., 2017) and the WAVE complex (Pipathsouk et al., 2021). Although components of CME enrich to bead-induced membrane invaginations in MDA-MB-231 cells (Elkhatib et al., 2017), clathrin LCa exhibited less robust recruitment to beads than WASP in neutrophils (Fig. 2 G). We previously found that the WAVE complex exhibits a preference for saddle curvatures and enriches to the necks of membrane invaginations and transepithelial holes (Pipathsouk et al., 2021). While the WAVE complex transiently enriched to some bead-induced invaginations, it failed to persistently enrich (Fig. 2 G), in contrast to WASP. Therefore WASP appears to be unusual among other curvature-sensitive proteins in its persistent marking of 500-nm-scale membrane invaginations in the front half of neutrophils.

**Figure S4.**
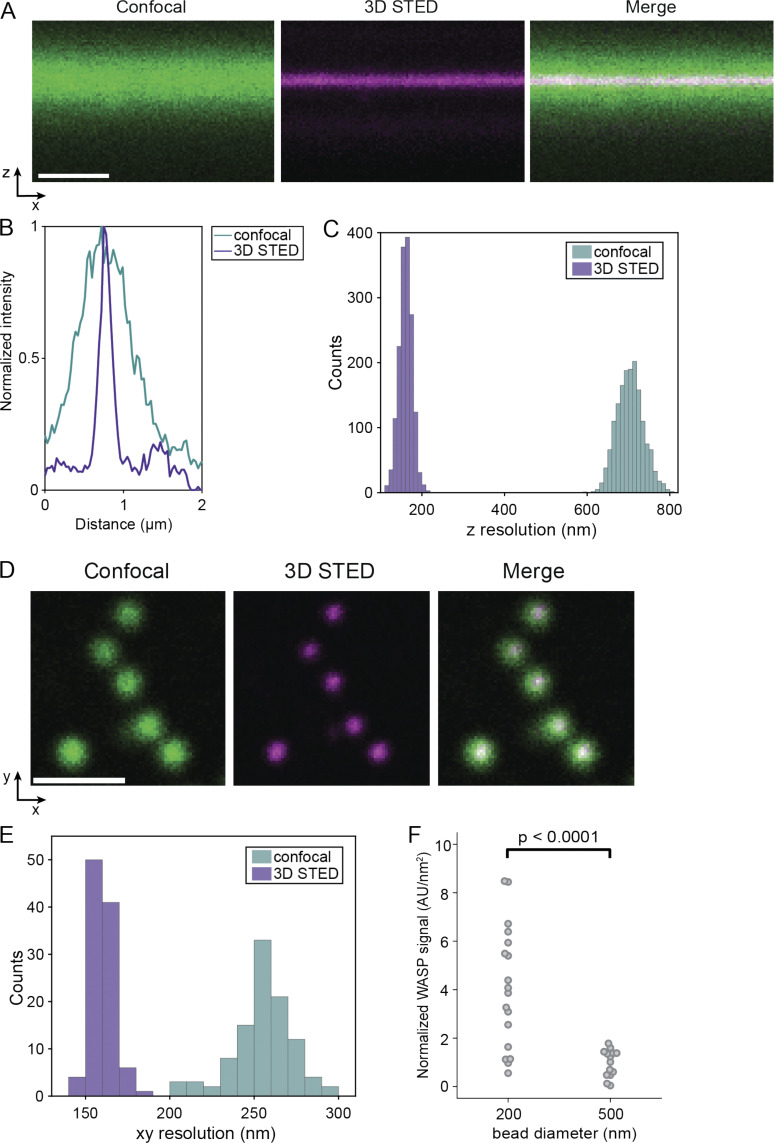
**Increased spatial and axial resolution achieved by 3D STED in HL-60 cells.** **(A)** Confocal (left), 3D STED (middle), and merged (right) image of an xz slice of the ventral membrane of an HL-60 cell labeled with CellMask Deep Red. Scale bar is 1 μm. **(B)** Representative fluorescence intensity profile along the axial direction in confocal (green) and 3D STED (magenta). **(C)** Histogram of axial resolution in confocal (green) and 3D STED (purple) imaging mode. 3D STED imaging provides a 4.4× increased axial resolution (160 ± 16 nm for 3D STED versus 704 ± 31 nm for confocal). The axial resolution is determined by the full-width half-maximum of a Gaussian fit to axial intensity profiles of the confocal and 3D STED images (*n* = 1,600 profiles). **(D)** Estimation of the lateral (xy) spatial resolution offered by 3D STED through imaging fluorescent beads (40 nm) in confocal (left) and 3D STED (right) mode with the same settings as in live-cell experiments. **(E)** Histogram of spatial resolution in confocal (green) and 3D STED (purple) imaging mode. 3D STED imaging provides a 1.6× increased spatial resolution (160 ± 6 nm for 3D STED versus 255 ± 17 nm for confocal). Spatial resolution is calculated by the full-width half-maximum of 2D Gaussian fits to the beads (*n* = 102 beads). Scale bar is 1 μm. **(F)** Integrated intensity of thresholded WASP signal across xyz stacks reveals significant enrichment of WASP to 200-nm beads compared with 500-nm beads (nonneck invaginations) when normalized to bead surface area. Mean normalized WASP intensity per unit area is 4.08 ± 0.59 a.u./nm^2^ for 200-nm beads and 0.97 ± 0.15 a.u./nm^2^ for 500-nm beads. P = 8.81E-05 by an unpaired two-tailed *t* test on the normalized WASP intensities at all beads. *n*_200 nm_ = 18 beads collected from two experiments and *n*_500 nm_ = 14 beads collected from one experiment. AU, arbitrary units.

**Figure 3.**
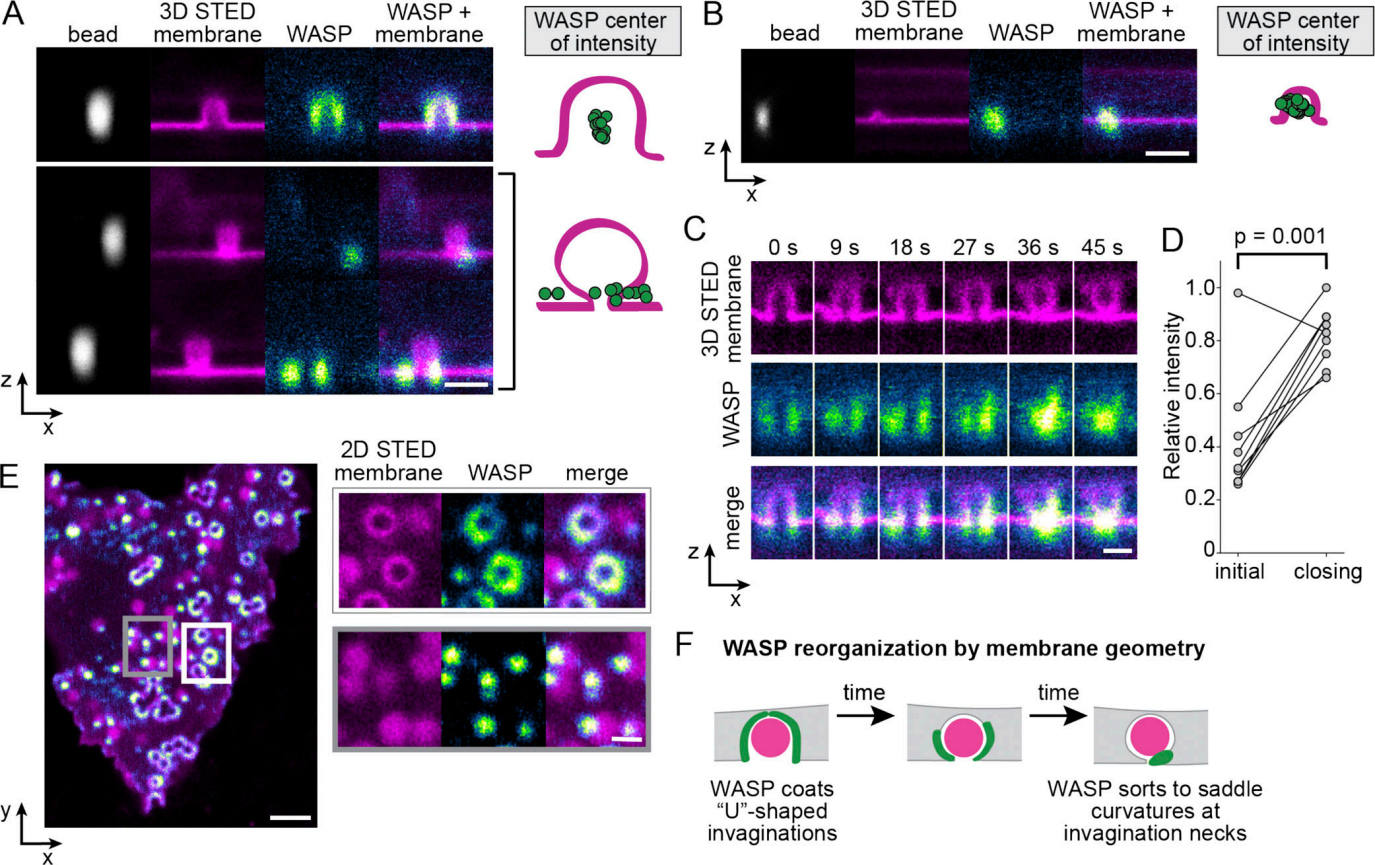
**Superresolution imaging reveals nanoscale reorganization of WASP by membrane geometry.** **(A)** Live-cell 3D STED microscopy of the membrane (labeled with CellMask Deep Red) coupled with confocal imaging of WASP on 500-nm beads show that WASP enriches to the site of highest inward curvature, either across the whole bud (top) or at an invagination neck when present (bottom). Right-hand side depicts the center of intensity of WASP for these two cases, with *n*_500nm, no neck_ = 17 and *n*_500nm, neck_ = 11 invaginations collected across eight and four cells, respectively, from the same experiment. Scale bar is 1 μm. **(B)** WASP enriches to the entire bud at smaller, 200-nm bead-induced invaginations, as reflected in the center of intensity of WASP to the right. *n*_200nm_ = 29 invaginations collected across nine cells from one experiment. Scale bar is 1 μm. **(C)** Time-lapse 3D STED imaging of the membrane reveals evolution from an open- to closed-neck structure at sites of bead-induced invagination. As this occurs, WASP redistributes and moves from the bead surface to the invagination neck. Scale bar is 500 nm. **(D)** As the membrane closes down around a bead, total WASP levels significantly increase. Mean normalized WASP intensity is 0.42 ± 0.08 a.u. at the first frame of the movie compared with 0.82 ± 0.04 a.u. at the time of neck closing on the single-invagination level. A paired two-tailed *t* test was used to compare the normalized intensity of WASP at these time points from nine invaginations collected across three experiments (P = 0.001). **(E)** 2D STED imaging of the cell membrane reveals both open and closed invaginations. At open invaginations, WASP coats the base (white box). As invaginations close down, WASP is reorganized into a focal accumulation at the neck (gray box). Scale bars are 2 μm and 500 nm in the inset. See Video 5 for the dynamics of this process using 2D STED. **(F)** Model for WASP evolution as the membrane of a cell constricts around a bead.

While we found that curvature informs WASP recruitment, it remained unclear what type of membrane geometry was driving this process, since bead-induced invaginations have both positive (inward) isotropic curvature (along the bead body) and saddle curvature (at the invagination neck). To elucidate WASP’s preferred curvature, we turned to live-cell 2D and 3D superresolution stimulated emission depletion (STED) microscopy of the membrane at bead-induced invaginations. Using 3D STED, we achieved a lateral (xy) resolution of 160 ± 6 nm and an axial (xz) resolution of 160 ± 16 nm (Fig. S4, A–E). We began with 500-nm beads to clearly separate the isotropically positive invagination body from the saddle-shaped neck. We observed two different classes of membrane organization around 500-nm beads, each of which correlated to distinct patterns of WASP enrichment (Fig. 3 A). In one set of cases, the membrane wrapped around a bead but failed to cinch around the bead bottom, forming an inverted U shape. In this case, WASP lined the entirety of the bead-induced membrane invagination (Fig. 3 A, top). In a second set of cases, the membrane formed a cinched neck under the bead, making an Ω shape (Fig. 3 A, bottom). When this morphology was observed, WASP no longer surrounded the entire structure but was instead concentrated onto the sides of the invagination neck. At smaller, 200-nm bead–induced invaginations, we could not resolve any membrane necks, and WASP organization at these sites appeared to match that of U-shaped invaginations (Fig. 3 B). However, others have reported saddle enrichment of N-WASP (Kaplan et al., 2020 *Preprint*) and the yeast orthologue Las17 (Mund et al., 2018) on this size scale using superresolution optical microscopy to image CME in fixed cells. By comparing WASP intensities at uniformly coated 200- and 500-nm bead–induced invaginations measured with 3D STED, we again found increased enrichment at beads with higher radii of curvature (Fig. S4 F).

We next investigated the temporal relation between the U and Ω invagination states, and using time-lapse 3D STED, were able to record U-shaped membranes closing around beads into Ω shapes (Fig. 3 C and Video 5). We found that not only does WASP reorganize from uniform coating to puncta during neck closing but also additional WASP is recruited to the invagination, highlighting a preference for the geometry found at the neck of 500-nm bead-induced invaginations (Fig. 3 D). Finally, when analyzing the ventral surface of cells with 2D STED, we see a higher proportion of U-shaped invaginations with uniform WASP closer to the cell front and Ω-shaped invaginations with punctate WASP further back (Fig. 3 E). These data indicate that over time, the membrane reconfigures from open neck to closed neck; during this transition, WASP is depleted from the bud of the invagination and concentrates to puncta in the neck of the invagination as more favorable neck geometries arise (Fig. 3 F).

**Video 5.** **Evolution of the membrane and WASP as cells run over beads (2D counterpart to ****Fig. 3 C****).** With time, the membrane closes around the bottom of the bead, transitioning from an open hole to a subresolution closed neck. At the same time, WASP redistributes from coating the open membrane hole to focal enrichment at the closed neck. Arrows mark examples where this occurs. WASP is imaged with confocal microscopy, and the membrane is imaged with 2D STED microscopy. Scale bar is 1 μm. Time, min:s. Video is displayed at 10 fps.

### Dual inputs of membrane curvature and cell polarity inform WASP recruitment

WASP is consistently recruited to sites of inward membrane deformation. However, if WASP enriched everywhere the membrane was invaginated by the substrate, cells might engage with the coverslip across their entire surface and be unable to productively migrate. We next investigated whether there was spatial information restricting where WASP can respond to curvature.

**Figure 4.**
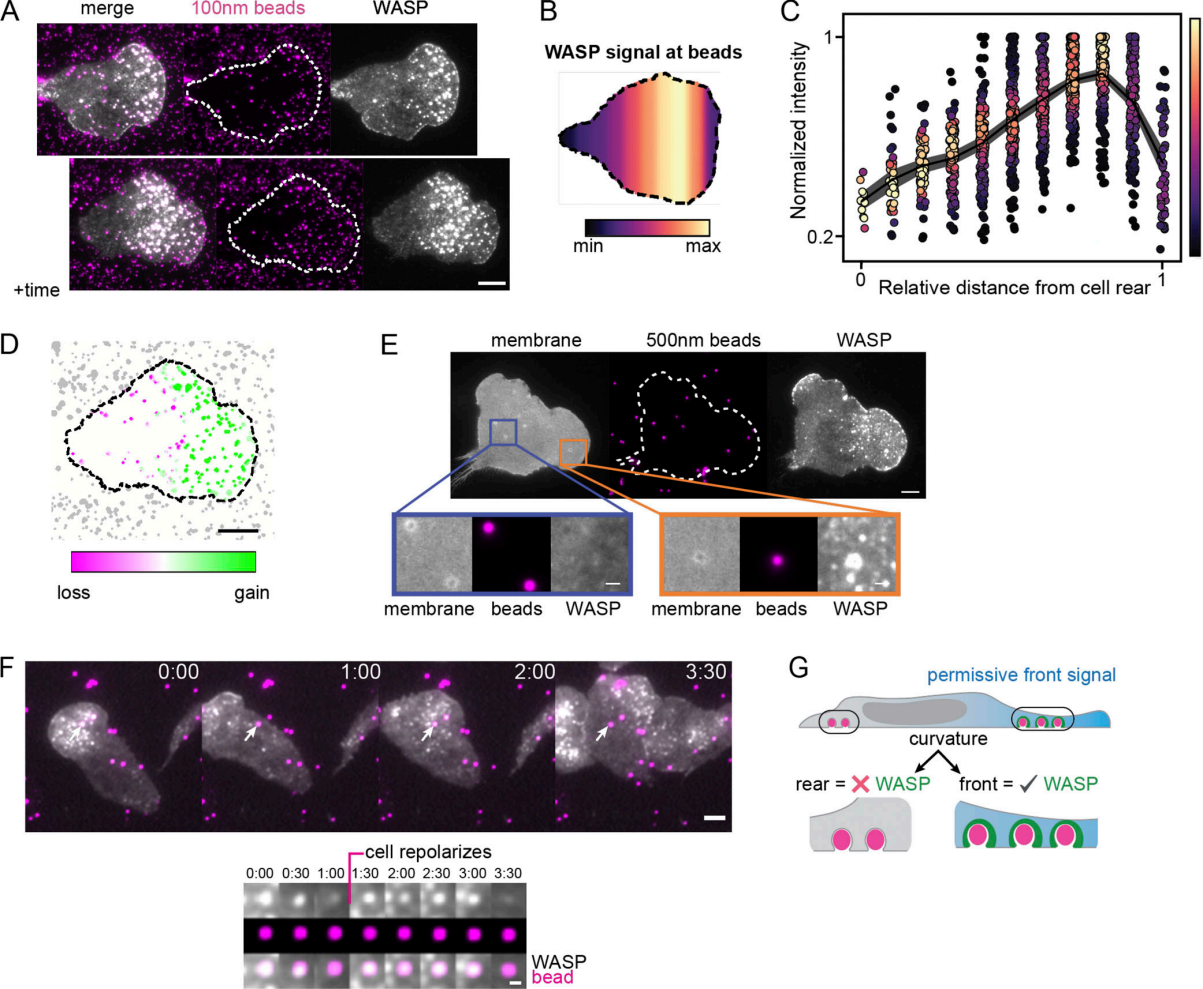
**WASP recruitment depends on both curvature and cell front signals.** **(A)** WASP enriches to bead-induced membrane deformations only in the front half of cells. Beads that have signal in the top montage lose signal as they move toward the cell rear. Images are ∼5 min apart. **(B)** Spatial distribution of average WASP signal from ∼135 beads that traverse the cell length. Data were collected across three experiments. The average WASP signal at a bead peaks in the front 20%–40% of the cell and then falls off despite continued presence of the bead under the cell. **(C)** Normalized traces of integrated WASP signal as a function of distance from the cell rear for the data in B. **(D)** Comparison of WASP signal gain and loss at beads from time point data in A. Beads at the cell front gain WASP (green), while beads at the cell rear lose WASP (magenta). **(E)** Membrane labeling (CAAX-tagBFP) confirms that beads continue to deform the membrane as they approach the cell rear yet no longer recruit WASP. Orange inset shows a bead at the front that deforms the membrane and recruits WASP, while the purple inset shows beads at the cell rear that deform the membrane but do not recruit WASP. Scale bar of the inset is 1 μm. **(F)** WASP signal diminishes as beads leave the front half of the cell, but reenriches at beads when front signals return. An example 200-nm bead is marked with an arrow and followed in the image series below. Scale bar of the inset is 1 μm. **(G)** Updated model to reflect that WASP requires both membrane curvature and front-localized signals for its enrichment. Scale bars are 5 μm unless otherwise specified. max, maximum; min, minimum.

**Figure S5.**
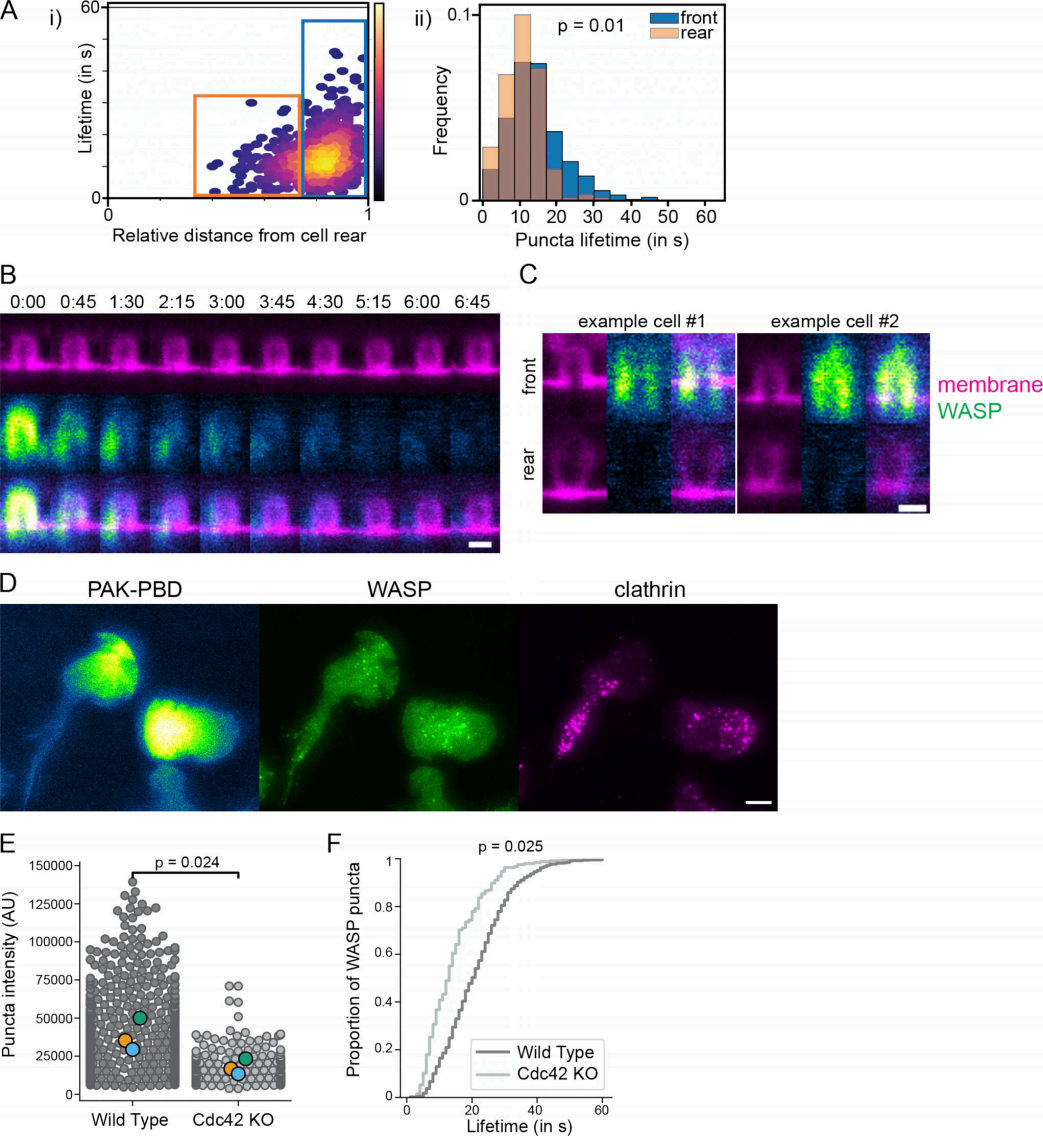
**Cell front signals underlie WASP puncta localization and dynamics.** **(A)** i: Scatter plot comparing the position of puncta appearance with lifetime reveals two populations (boxed). ii: Histograms of the boxed populations show puncta that appear closer to the cell rear extinguish faster (orange bars), suggesting that position within the cell influences puncta disappearance. More than 650 puncta were collected across 26 cells from three experiments. Mean lifetime is 14.3 ± 0.9 s for puncta that nucleate in the front 25% of the cell (blue box, *n* = 486 puncta) and 10.6 ± 0.25 s for puncta that nucleate farther back (orange box, *n* = 172 puncta). P = 0.01 by a paired two-tailed *t* test on replicate means. **(B)** Time-lapse STED imaging shows that membrane invaginations maintain geometry but lose WASP signal as the cell continues moving. Scale bar is 500 nm. **(C)** Single xz STED slices of beads at the cell front and cell rear of the same cell show that despite similar geometry, WASP is only recruited to invaginations at the front. Images are scaled to the same intensity. Scale bar is 500 nm. **(D)** WASP colocalizes with the canonical front marker PAK-PBD that reads out active Rac (Weiner et al., 2007). Both WASP and PAK-PBD fail to overlap with clathrin LCa. Scale bar is 5 μm. **(E)** Comparison of WASP puncta intensity in endogenously tagged WT and Cdc42 KO backgrounds. In addition to being significantly fewer in number in Cdc42 KO cells (Fig. 5 F), the WASP puncta that do form are less than half as bright in the absence of Cdc42. *n*_puncta, WT_ = 1,211 and *n*_puncta, Cdc42 KO_ = 247, which were collected across 46 and 40 cells, respectively. P = 0.024 by a paired two-tailed *t* test on replicate means. **(F)** Cumulative distribution function of lifetime of puncta in E. WASP puncta lifetime in Cdc42-null cells is 30% less. P = 0.025 by a paired two-tailed *t* test on replicate means. AU, arbitrary units.

Established curvature-sensitive proteins typically enrich to a given plasma membrane morphology irrespective of their physical location in the cell (Zhao et al., 2017; Lou et al., 2019). Strikingly, WASP only enriched to beads in the front half of the cell (Fig. 4 A). When a cell ran into a bead, WASP was recruited within seconds (Fig. 2 B). Then, as the cell migrated over the bead and it progressed to the cell rear, WASP signal was lost (Fig. 4, B–D). The disappearance of WASP correlated more strongly with puncta position than duration, as puncta that nucleated further from the cell front exhibited shorter lifetimes (Fig. S5 A). To ensure that WASP disappearance was not a consequence of poor membrane deformation at the cell rear, we imaged the membrane and confirmed that 500-nm bead–induced membrane deformations persisted at the cell rear but lost WASP signal (Fig. 4 E). Additionally, using 3D STED, we observed that bead-induced invaginations lost WASP over time, as they moved toward the cell rear (Fig. S5 B). Invaginations in the front half of the cell were consistently WASP positive, while invaginations at the rear of the same cells did not have WASP, despite their similar membrane geometries (Fig. S5 C). Interestingly, we found that WASP signal could be restored to beads that had exited the front permissive zone if the cell repolarized and front signals were restored, which occurs during processes like turning (Fig. 4 F). This corequirement of cell front signals and curvature support an AND gate logic for WASP recruitment (Fig. 4 G).

**Figure 5.**
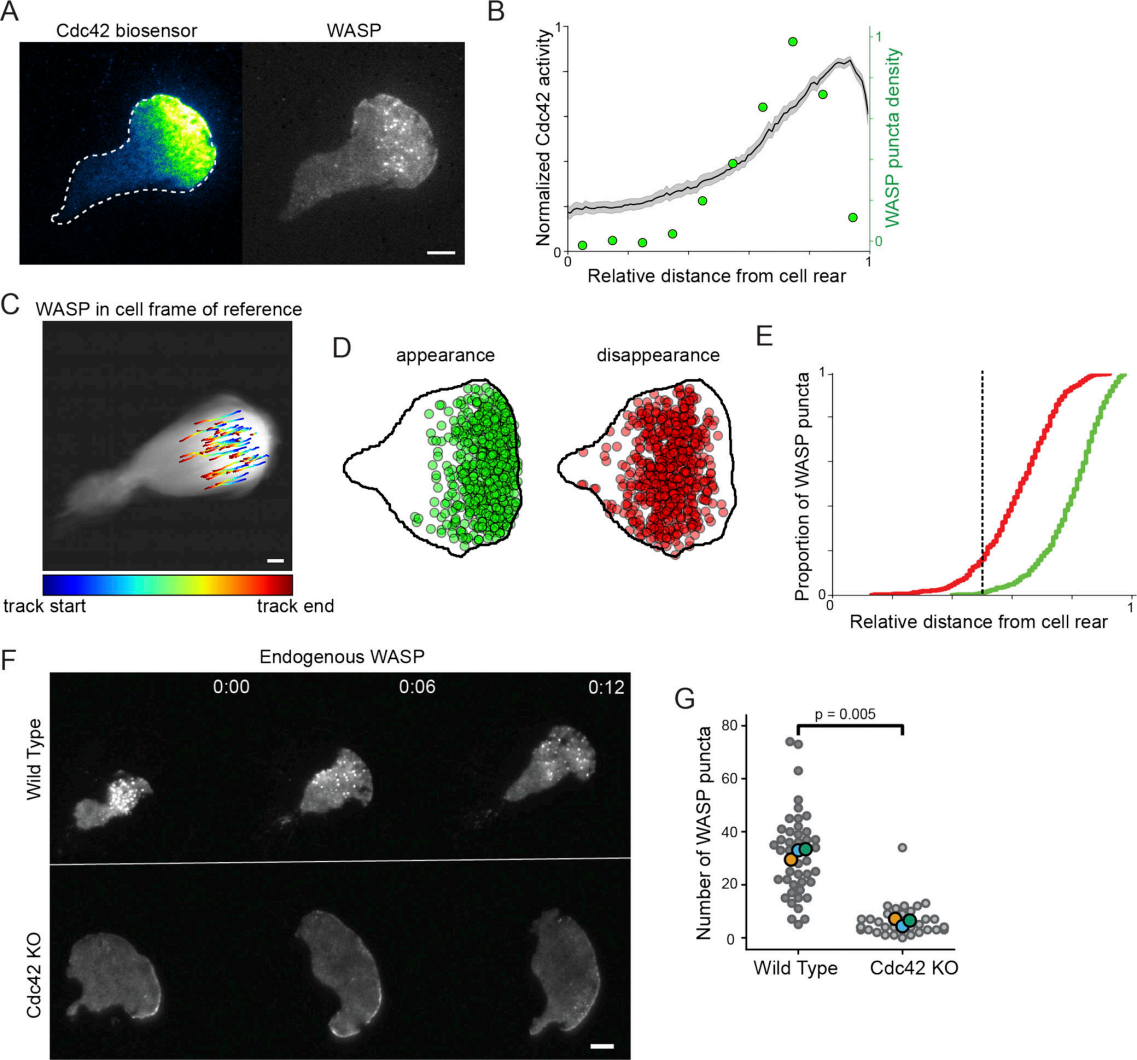
**A polarized upstream GTPase, Cdc42, sets a permissive zone for ventral WASP puncta formation.** **(A)** Endogenously tagged WASP forms puncta that overlap with regions of active Cdc42 (assayed via TagRFP-T–conjugated WASP-GBD; Nalbant et al., 2004). **(B)** Cdc42 activity exists as a gradient across the ventral surface of polarized HL-60s that extends across the front half of the cell as previously observed in Yang et al. (2016). Biosensor data are an average normalized line scan along the major axis of the cell for 49 cells collected over three experiments. Overlaid is the WASP puncta distribution presented in Fig. 1 D. **(C)** Trajectories of single WASP puncta rendered in the cell’s frame of reference show that puncta nucleate at the cell front and extinguish around half the cell length. Trajectories are overlaid onto the summed frames of a registered cell migrating over 40 s and are colored according to relative temporal position within a given puncta’s total duration. See also Video 6. **(D)** Positions of WASP puncta appearance and disappearance mapped onto a representative cell shape support this front bias across many cells. Positions were recorded for >650 puncta from 26 cells in three experiments. **(E)** Cumulative distribution functions for relative puncta appearance (green) and disappearance (red) positions show that the majority of events occur before one half of the cell length (vertical dashed line). **(F)** Endogenous WASP distributions in WT and Cdc42 KO cells reveal a loss of ventral WASP puncta in the absence of Cdc42. Images are displayed on the same intensity scale. **(G)** Number of WASP puncta per cell in WT and Cdc42 KO cells. *n*_WT_ = 46 and *n*_KO_ = 40 cells collected across three replicates. P = 0.005 by a paired two-tailed *t* test on replicate means. Scale bars are all 5 μm.

Due to the apparent dependence of WASP enrichment to bead-induced invaginations on cell front signals, we sought to establish whether the spatial distribution of natively formed WASP puncta overlap with canonical front signals in neutrophils. Indeed, the spatial distribution of WASP puncta overlapped with biosensor activity for the front-polarized GTPases Cdc42 (assayed via the GBD [GTPase-binding domain] of WASP; Fig. 5, A and B; Kim et al., 2000; Nalbant et al., 2004; Levskaya et al., 2009) and Rac (assayed via the PBD [p21-binding domain] of PAK1 [p21-activated kinase 1] ; Fig. S5 D). Notably, WASP puncta were shifted relative to GTPase activity, potentially indicating a delay between GTPase activation and WASP recruitment.

In addition to the spatial distribution of WASP puncta, we also explored puncta dynamics. By remapping microscopy data to the cell’s reference frame, we were able to track the position of WASP puncta appearance and disappearance relative to the cell front. This analysis confirmed that puncta persist only in the front half of the cell (Fig. 5 C and Video 6). Specifically, WASP puncta nucleate at 19.8 ± 0.4% and extinguish by 37.4 ± 0.5% of the cell length on average (Fig. 5 D). Ultimately, 84% of all puncta disappear by 50% of the cell length, and 95% disappear by 60%, suggesting a coupling between WASP puncta dynamics and their physical position in the cell (Fig. 5 E).

**Video 6.** **WASP puncta dynamics in the cell’s frame of reference (as in ****Fig. 5 C****).** A subset of puncta was tracked from nucleation to extinction. Puncta nucleate in the cell front and disappear around half of the cell length. Trajectories shown in the right panel are colored according to temporal position within the given puncta’s total duration. Scale bar is 5 μm. Time, min:s. Video is displayed at 6 fps.

To investigate how WASP integrates cell polarity inputs with its curvature-sensitive membrane enrichment, we wanted to better understand the signals that specify the region permissive for WASP recruitment. WASP/N-WASP is known to link Cdc42 to Arp2/3 complex activation (Rohatgi et al., 1999; Higgs and Pollard, 2000), and Cdc42 activity is polarized toward the front of migrating neutrophils (Yang et al., 2016). In Cdc42-null cells, endogenous WASP failed to form its front-biased ventral punctate distribution (Fig. 5, F and G). Instead, WASP exhibited a higher enrichment to the tip of the leading edge (Fig. 5 F). When ventral puncta did manage to form, they were both significantly dimmer and shorter lived (Fig. S5, E and F), supporting a significant role for Cdc42 as an input to WASP ventral puncta formation, though other signals appear to regulate the pool of WASP at the tip of lamellipodia. In other settings, WASP localization does not depend on direct GTPase binding (Tal et al., 2002; Amato et al., 2019).

Our data show that WASP puncta formation and curvature sensitivity occur only in a permissive, front-polarized region. WASP identifies membrane invaginations as they form at the cell front, and WASP dissociates from the invaginations around the time they cross the cell midline. We next investigated how this polarization impacts the cytoskeleton.

### WASP links membrane topology with the actin cytoskeleton

**Figure 6.**
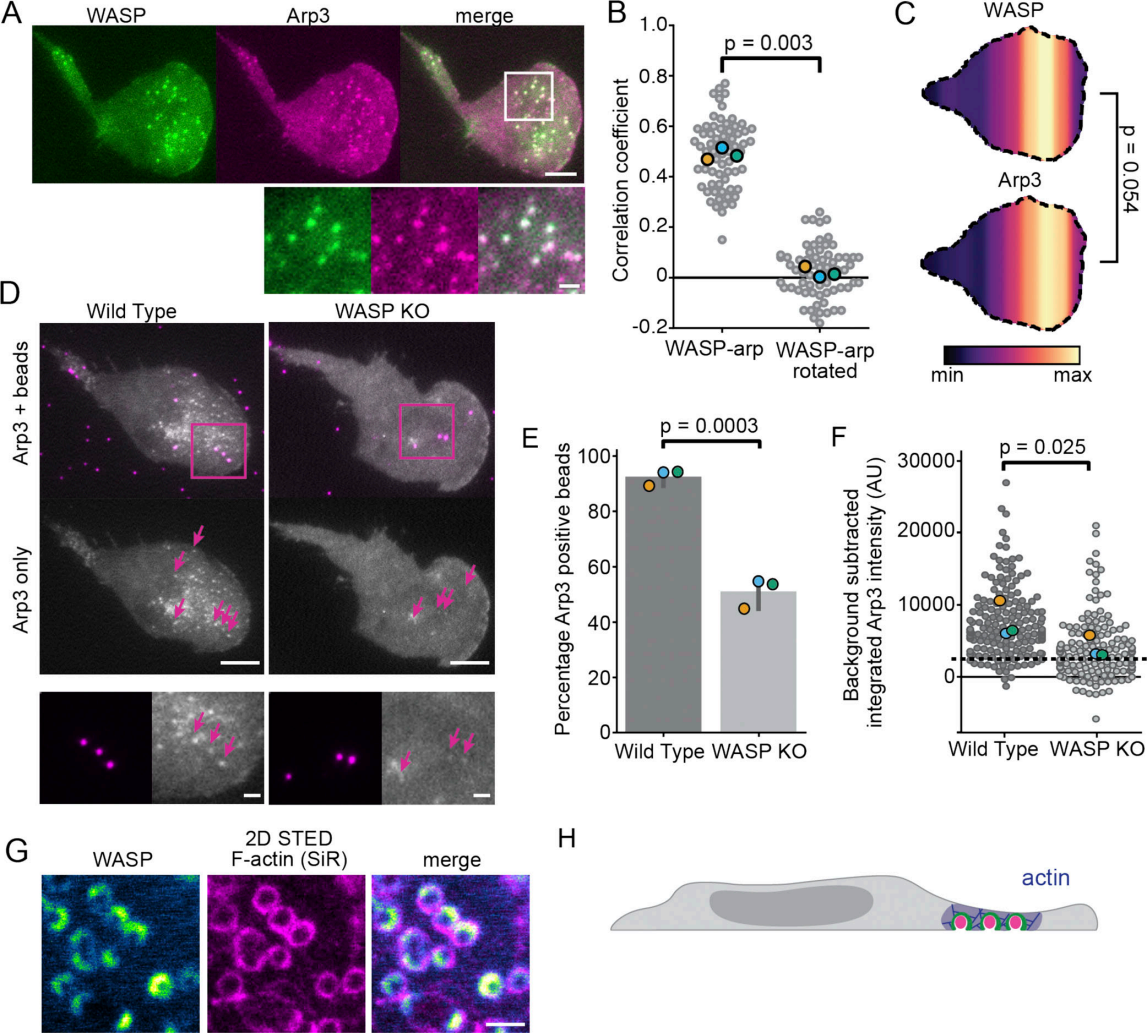
**WASP mediates actin polymerization at sites of membrane deformation.** **(A)** Endogenous WASP colocalizes with TagRFP-T-Arp3. Scale bars are 5 μm and 1 μm in the inset. **(B)** Pearson correlation coefficients of WASP and Arp3 show significant colocalization (*r* = 0.49 ± 0.01) compared with the correlation coefficients between WASP and a 90° rotation of the Arp3 channel (0.02 ± 0.02). *n* = 68 7.5 × 7.5–μm regions of interest collected across three experiments. P = 0.003 by a paired two-tailed *t* test on replicate means, which are denoted throughout the figure by enlarged markers. **(C)** Spatial distributions of WASP and Arp3 puncta reveal similar organization of these proteins. *n* = 18 coexpressing cells collected across three experiments with *n*_WASP_ = 559 and *n*_Arp3_ = 417 total puncta. The mean relative puncta position is 0.64 ± 0.02 for WASP and 0.65 ± 0.02 for Arp3, with the cell rear defined as 0 and the cell front as 1. P = 0.054 by a paired two-tailed *t* test on the mean puncta position of each marker in each cell. **(D)** WASP KO cells fail to recruit appreciable amounts of the Arp2/3 complex to 100-nm bead-induced invaginations. Arrows denote the position of beads that are under the cell front. Below is a zoomed inset of the boxed region. All images are scaled equally. Scale bars are 5 μm and 1 μm in the inset. **(E)** Only about half of the beads (51.3 ± 3.1%) under the cell front in WASP KO cells are able to form Arp2/3 puncta (determined by the ability to fit a Gaussian to Arp3 signal at a bead) compared with 93.7 ± 1.7% of beads for WT cells. *n*_WT_ = 172 beads and n_KO_ = 130 beads collected across three experiments. P = 0.0003 by an unpaired two-tailed *t* test on replicate means. **(F)** Arp2/3 complex recruitment to bead-induced invaginations is significantly less in WASP KO cells than in WT cells. For each bead, the integrated intensity of background-subtracted Arp3 signal was calculated for the brightest Arp3 frame. The dashed line marks the approximate threshold needed to fit a Gaussian in panel E. Measurements below this line are close to background or noise. The mean intensity of Arp3 at beads is 7,696 ± 1,454 a.u. in WT cells and 4,020 ± 886 a.u. in WASP KO cells on the replicate level. *n*_WT_ = 164 and *n*_KO_ = 143 beads collected across three experiments. P = 0.025 by a paired *t* test on replicate means. **(G)** WASP and F-actin (reported with SiR-actin) colocalize at bead-induced membrane invaginations in the front half of the cell. Scale bar is 1 μm. **(H)** Updated model to reflect front-biased WASP-dependent actin polymerization. AU, arbitrary units; max, maximum; min, minimum.

To determine whether actin polymerization occurs at WASP puncta, we transduced WASP KI cells with a TagRFP-T-tagged Arp3 subunit of the Arp2/3 complex. Arp3 colocalizes with endogenous WASP puncta in the front half of the cell (Fig. 6, A and B). Unlike in CME in mammalian cells where N-WASP signal peaks roughly 10 s before that of Arp3 (Taylor et al., 2011), we were unable to resolve a temporal offset between recruitment of WASP and Arp3 at puncta. This observation is, however, consistent with measurements made for WASP and the Arp2/3 subunit ArpC4 in *Dictyostelium discoideum* (Amato et al., 2019). Additionally, in comparing the spatial distributions of WASP and Arp3 puncta across the cell length, we find significant overlap between the signals (Fig. 6 C). Given these observations, we conclude that Arp3 behavior closely mirrors that of WASP at puncta. Enrichment of an actin nucleator to these sites suggests that polymerization is occurring and that native WASP puncta are F-actin–rich structures.

Next, we sought to determine whether actin polymerization occurs at bead-induced invaginations and, if so, whether this process depends on WASP. Plating our Arp3-tagged cells onto beads, we found that the Arp2/3 complex, like WASP, enriches to nearly all bead-induced invaginations while they are in the front half of the cell (Fig. 6 D). However, repeating these measurements in WASP KO HL-60s showed an ∼50% reduction in the number of beads that induced Arp2/3 puncta formation and a significant decrease in the integrated Arp3 intensity at beads despite similar expression levels in both backgrounds (Fig. 6, D–F). Additionally, while there persisted a band of Arp3 signal immediately behind the leading edge, Arp3 signal across the rest of the cell was quite uniform, and puncta outside those formed at beads were rare (Fig. 6 D). Therefore, a significant pool of Arp2/3 complex recruitment to sites of inward membrane deformation depends on WASP, though the residual pool of Arp2/3 enrichment indicates that other NPFs can participate as well.

Finally, we used the live-cell actin probe SiR-actin to assay for F-actin at bead-induced invaginations and found colocalization of SiR-actin with WASP at beads under the front half of the cell (Fig. 6, G and H). Our finding that WASP plays an important role in linking membrane deformation to the actin cytoskeleton led us to next investigate how WASP-dependent actin structures affect neutrophil migration.

### Integration of topographical features during neutrophil migration depends on WASP

Given the clear in vivo phenotype of WASP KO neutrophils (Snapper et al., 2005; de Noronha et al., 2005; Westerberg et al., 2005; Jones et al., 2013) and our observation that WASP recruitment is regulated by inward membrane curvature, we hypothesized that WASP-dependent migration may be particularly acute for cells challenged by a complex, membrane-deforming environment. To assess this, we probed the role of WASP during migration on 800 × 800–nm nanoridged substrates designed to mimic the geometry of aligned collagen fibers cells encounter in vivo (Ray et al., 2017).

**Figure 7.**
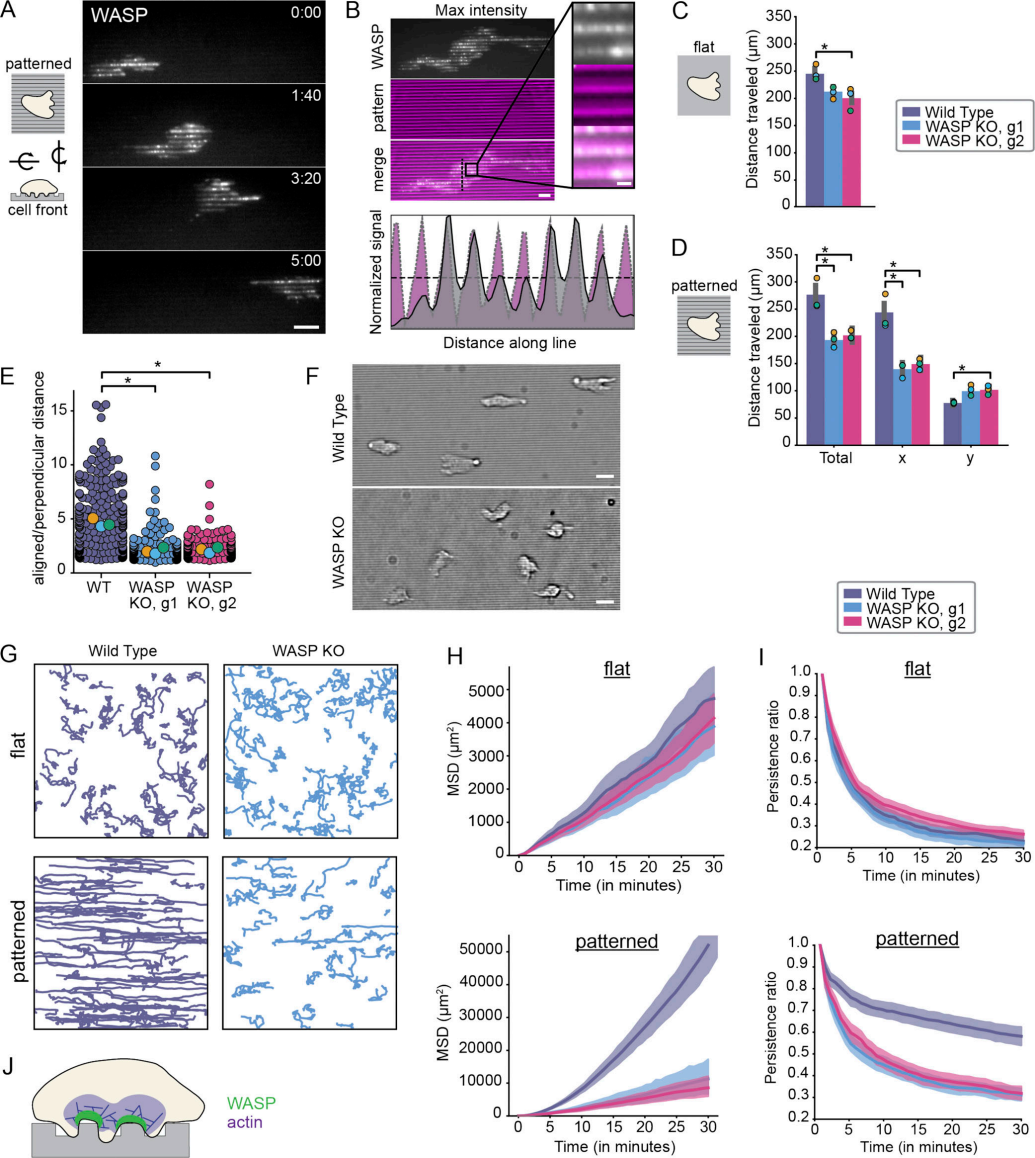
**WASP** **KO cells are defective in their ability to interpret the topological features of their environment.** **(A)** WASP puncta enrich at nanoridges as cells migrate over patterns. Scale bar is 10 μm. **(B)** Maximum intensity projection of data in A shows that WASP localizes to nanoridge peaks. An intensity profile for WASP and the pattern along the vertical line in the merged image is shown below. Scale bar is 5 μm and 1 μm in the inset. **(C)** There is a modest difference in the total distance traveled by WT (purple) and WASP KO (guide 1 [g1] = blue, g2 = magenta) cells plated on flat substrates. *n*_WT_ = 253 cells, *n*_KO1_ = 215 cells, and *n*_KO2_ = 181 cells collected across three experiments. Total distance traveled: WT = 246 ± 1 μm, WASP KO1 = 210 ± 6 μm, and WASP KO2 = 201 ± 12 μm on the replicate level. Replicate means are denoted throughout the figure by enlarged markers. Only one of the WASP KO clones was significantly different from WT. For this case, P = 0.026 by a paired two-tailed *t* test on replicate means. In the other case, P = 0.13. See also Video 7. **(D)** The total distance traveled by WASP KO cells is significantly less than that of WT cells on nanoridged substrates due to defective movement along the patterns (here, the x axis). *n*_WT_ = 188 cells, n_KO1_ = 172 cells, and n_KO2_ = 242 cells collected across three experiments. Total distance traveled: WT = 273 ± 17 μm, WASP KO1 = 194 ± 8 μm, and WASP KO2 = 202 ± 5 μm on the replicate level. Total distance traveled in x: WT = 241 ± 19 μm, WASP KO1 = 139 ± 8 μm, and WASP KO2 = 149 ± 6 μm on the replicate level. Total distance traveled in y: WT = 78 ± 1 μm, WASP KO1 = 102 ± 6 μm, and WASP KO2 = 102 ± 5 μm on the replicate level. *, P < 0.05, which was determined by a paired two-tailed *t* test on replicate means. Unless labeled, the difference is not significant. P_total, WT/KO1_ = 0.018, P_total, WT/KO2_ = 0.028, P_x, WT/KO1_ = 0.023, P_x, WT/KO2_ = 0.023, P_y, WT/KO1_ = 0.061, P_y, WT/KO2_ = 0.035. See also Video 8. **(E)** Ratio of aligned to perpendicular migration reported in D highlights defective nanopattern sensing by WASP KO cell lines. Average ratio of total aligned to total perpendicular distance traveled: WT = 4.02 ± 0.23, WASP KO1 = 1.47 ± 0.17, and WASP KO2 = 1.57 ± 0.15 on the replicate level. *, P < 0.05, which was determined by a paired two-tailed *t* test on replicate means. P_WT/KO_ = 0.012 and P_WT/KO2_ = 0.0078. **(F)** Bright field images reveal clear alignment of WT cells to horizontal nanoridges, while WASP KO cells largely failed to align. Scale bar is 5 μm. **(G)** WT and WASP KO cell trajectories over 1 h for cells migrating on flat substrates (top) and nanoridged substrates (bottom). Image dimensions are ∼665 × 665 μm. **(H)** The MSDs of WT and WASP KO cells are indistinguishable on flat substrates (top), while the MSDs of WASP KO cells are significantly less than that of WT cells on nanoridged substrates (bottom). Shading denotes a 95% confidence interval. Trajectories are the same as those assayed in C and D, respectively. Of note, the MSD of WT cells is 10-fold greater on nanoridged substrates than on flat substrates. **(I)** The persistence ratios of WT and WASP KO cells are indistinguishable on flat substrates (top), while the persistence ratios of WASP KO cells are significantly less than that of WT cells on nanoridged substrates (bottom). Shading denotes a 95% confidence interval. Trajectories are the same as those assayed in C and D, respectively. **(J)** Model of the role of WASP and WASP-mediated actin polymerization in topology sensing and contact guidance.

First, we tested whether WASP puncta form at sites of inward curvature generated by nanoridges as they did at beads (Fig. 2) and on collagen fibers (Fig. S2, B and C). Indeed, WASP puncta accumulate on nanoridges where the pattern pushes into the cell (Fig. 7, A and B). This behavior was also recently observed for WASP in dendritic cells (Gaertner et al., 2021* Preprint*). Unlike focal adhesions that form on both the ridges and the grooves of these patterns (Ray et al., 2017), WASP localized specifically to the positive (inward) curvature-inducing ridges. Additionally, despite long stretches of contact between cells and the patterns, WASP enrichment remained punctate and failed to continuously localize across ridges under the cell. These data suggest that WASP may have an intrinsic preference for focal distributions, reminiscent of the condensate-like behavior of N-WASP oligomerization (Li et al., 2012; Banjade and Rosen, 2014; Case et al., 2019).

To determine whether WASP enrichment on nanoridges reflected a role in migration, we next compared the effect of flat versus textured substrates on the migration of WT and WASP KO HL-60s (Fig. S1, F and G). There was a modest difference between the total distance traveled by WT and WASP KO cells on flat substrates (15%–19% decrease; Fig. 7 C and Video 7). In contrast, WASP KO cells plated on nanoridges exhibited a greater reduction in total distance traveled compared with WT cells (27%–30% decrease; Fig. 7 D and Video 8). When we decomposed cell movement into axes that aligned with or were perpendicular to the nanopatterns, the observed migration defect was explained by a decrease in the ability of WASP KO cells to orient their migration along patterns. In fact, WASP KO cells moved ∼60% less than WT cells in the direction of the patterns while moving ∼30% more than WT cells in the direction perpendicular to the patterns. This is further reflected by a large difference in the ratio of aligned to perpendicular migration between WT and WASP KO cells (Fig. 7 E). The observed increase in perpendicular migration as opposed to a decrease in migration along both axes highlights a defect in the ability of WASP KO cells to sense and respond to the nanopatterned features of their substrate rather than to a general defect in cell motility. Indeed, WT HL-60 cells largely aligned with the ridges, while WASP KO cells did so with much lower frequency (Fig. 7 F and Video 8). WT cells also moved more persistently along ridges and sometimes followed them across the entire field of view (>665 μm; Fig. 7 G, bottom left). In contrast, persistent migration along nanoridges occurred less frequently in WASP KO cells (Fig. 7 G, bottom right).

**Video 7.** **WASP KO HL-60 cells exhibit relatively minor migration defects on flat substrates (as in ****Fig. 7, C and G–I****, top).** Cells are labeled with NucBlue for tracking and imaged for 1 h at 37°C in 5% CO_2_. Overlaid are cell trajectories that last at least half the movie. Track color represents frame number. Scale bar is 10 μm. Time, min:s. Video is displayed at 10 fps.

**Video 8.** **WASP KO HL-60 cells exhibit significant migration defects on textured (800 nm nanoridged) substrates (as in ****Fig. 7, D–F; and Fig. 7, G–I****, bottom).** Cells are labeled with NucBlue for tracking and imaged for 1 h at 37°C in 5% CO_2_. Overlaid are cell trajectories that last at least half the video. Track color represents frame number. Scale bar is 10 μm. Time, min:s. Video is displayed at 10 fps.

To better understand how WT and WASP KO cells differentially explore space on flat versus patterned substrates, we calculated the mean squared displacement (MSD) for each cell type in these environments. In the absence of patterns, the MSDs of WT and WASP KO cells were largely indistinguishable (Fig. 7 H, top). However, on nanoridges, the MSD was significantly larger for WT cells than WASP KO cells across increasing time offsets (Fig. 7 H, bottom). Due to an apparent difference in persistence between WT and WASP KO cells on nanoridges (Fig. 7 G, bottom; and Video 8), we also calculated a persistence ratio, defined here as the ratio between displacement from the starting position and total distance traveled. In the absence of patterns, the persistence ratio steadily falls off over increasing time windows for both WT and WASP KO cells, since there is no input biasing the direction of movement (Fig. 7 I, top). Conversely, in the presence of nanoridges, WT cells are significantly more persistent than WASP KO cells on both short and long time scales (Fig. 7 I, bottom).

Taken together, these observations reveal an essential role for WASP in substrate topology sensing and contact guidance in neutrophils (Fig. 7 J).

## Discussion

Neutrophils need to navigate complex 3D paths through dense extracellular matrices in vivo (Lämmermann et al., 2013). To move in this environment, a cell must be able to sense its physical surroundings and coordinate its movement with the features of its substrate. Our work suggests that WASP plays a role in mediating this process. First, WASP responds to the topology of the substrate by enriching to sites of inward, substrate-induced membrane deformation (Figs. 2 and 3). WASP then facilitates recruitment of the Arp2/3 complex for local actin assembly, thereby coupling substrate features with the cytoskeleton (Fig. 6). WASP functions as a dual integrator of cell front signals and membrane curvature, enriching to membrane invaginations only in the front half of the cell (Figs. 4 and 5). To our knowledge, this is the first report of curvature sensitivity that is informed by physical location within a cell. Through these characteristics, WASP is able to (1) identify substrate features on which to anchor the cell, (2) pattern actin polymerization to leverage substrate contact sites for locomotion, and (3) constrain substrate engagement to regions that yield productive migration. As a consequence of these features, WASP KO neutrophils are particularly defective at coordinating substrate topology with cell guidance (Fig. 7).

While the importance of WASP has long been appreciated due to its role in Wiskott–Aldrich syndrome, its contribution to neutrophil migration has been unclear. In animal models of this disease, neutrophils are defective at homing to sterile wounds (Jones et al., 2013) and sites of infection (Snapper et al., 2005; Kumar et al., 2012). However, in vitro WASP KO neutrophils have shown mixed phenotypes. Some studies have reported defective migration (Ochs et al., 1980; Fritz-Laylin et al., 2017), while others have reported no defect (Zicha et al., 1998; Zhang et al., 2006). One possible reason for this discrepancy is our finding that WASP is primarily used during migration in complex environments where substrate topology sensing and engagement play a critical role.

Much of what we know about WASP function has been inferred from that of its ubiquitously expressed homologue N-WASP, which plays a key role in CME (Merrifield et al., 2004; Benesch et al., 2005; Kessels and Qualmann, 2002), among other functions (Isaac et al., 2010; Nusblat et al., 2011; Mizutani et al., 2002; Yamaguchi et al., 2005; Yu et al., 2012; Yu and Machesky, 2012; Zhang et al., 2006; Misra et al., 2007; Liu et al., 2013). Whether WASP plays a similar role in immune cells was not known. Our work reveals that a critical function of WASP in neutrophils is divergent from the canonical functions of N-WASP. However, we find some parallels between WASP and N-WASP; both proteins use membrane invagination to locally polymerize actin via Arp2/3 complex recruitment. This process culminates in CME for N-WASP, but not WASP, where the function appears to be in coupling the actin cytoskeleton to substrate-induced membrane deformations that guide cell movement. This is not the first example of cells repurposing the curvature sensitivity of the endocytic machinery to help engage with their substrate. In particular, collagen fibers induce persistent nonendocytic clathrin-coated structures that aid in 3D migration in other cellular contexts (Elkhatib et al., 2017). We propose WASP is performing a similar function in highly motile neutrophils.

WASP and N-WASP have also been implicated in building podosomes and invadopodia (Isaac et al., 2010; Nusblat et al., 2011; Mizutani et al., 2002; Yamaguchi et al., 2005; Yu et al., 2012; Yu and Machesky, 2012), which are membrane exvaginations that participate in extracellular matrix degradation and mechanosensation (Albiges-Rizo et al., 2009; Murphy and Courtneidge, 2011). Instead of degrading extracellular matrix, neutrophils squeeze through pores in collagen meshworks, which may rely on an alternate mode of substrate engagement (Lämmermann et al., 2008, 2013; Steadman et al., 1997; Allport et al., 2002). The WASP puncta we observe wrap around the substrate, instead of poking into it. It is interesting that these structures are morphologically distinct but use homologous proteins in their construction. Although invadopodia and podosomes are ultimately protrusive, it is possible that they are initiated in response to inward membrane deformations. Indeed, invadopodia concentrate their actin polymerization and matrix degradation at collagen fibers that indent the cell and impede cell movement (Ferrari et al., 2019), and podosomes only become exvaginations upon actomyosin contraction (Labernadie et al., 2010) and may be initiated through other membrane deformations. In dendritic cells, WASP stimulates focal actin-based protrusions in response to local compression (Gaertner et al., 2021 *Preprint*). A response to membrane invaginations may represent a shared feature of these cellular structures despite the differences in their ultimate morphology.

WASP is unusual in that it is able to integrate both cell front signals and membrane curvature for its recruitment; only membrane deformations in the front half of the cell result in WASP enrichment (Fig. 4; and Fig. S5, B and C). How does WASP sense cell polarity? A promising candidate is Cdc42; it is one of the primary inputs to WASP-mediated actin assembly (Rohatgi et al., 1999; Higgs and Pollard, 2000), and Cdc42 activity is polarized toward the front of migrating neutrophils (Yang et al., 2016). Indeed, we show that Cdc42 is required for the front-biased ventral punctate distribution of WASP (Fig. 4, E and F). This dependence may either be through direct binding of WASP to Cdc42 via its Cdc42- and Rac-interactive binding domain (Aspenström et al., 1996) or through interactions with bridging proteins that bind both Cdc42 and WASP (Ho et al., 2004).

Overall, our work establishes WASP as a link between cell movement and substrate topology. Previous reports have found that different cell types, including HL-60s, can sense nanopatterned topologies and respond to them through reorientation and aligned migration (Driscoll et al., 2014; Sun et al., 2015). In the case of adherent cells, this is thought to be through imposing spatial constraints on focal adhesion formation and maturation (Ray et al., 2017). However, for loosely adherent cells like neutrophils and *D. discoideum* that do not rely on long-lived adhesive structures (Lämmermann and Sixt, 2009; Driscoll et al., 2014), the molecular mechanism unpinning physical environment sensing and alignment to substrate topologies has remained unclear. Some proposed mechanisms for cytoskeletal remodeling in response to membrane deformation include membrane geometry sensing (Itoh et al., 2005; Zhao et al., 2017; Lou et al., 2019; Takenawa and Suetsugu, 2007), membrane tension sensing (Houk et al., 2012; Tsujita et al., 2015; Diz-Muñoz et al., 2016), force adaptation (Bieling et al., 2016; Mueller et al., 2017), and even actin filament bending (Risca et al., 2012). The fact that WASP is both curvature sensitive and essential for HL-60 alignment to nanoridges supports WASP-mediated actin polymerization as a mechanism by which loosely adherent cells can sense and align their shape and movement with their substrate’s topology.

Membrane curvature has emerged as a key regulator for WASP family proteins; other NPFs from this family, such as N-WASP (Lou et al., 2019) and WAVE (Pipathsouk et al., 2021), also preferentially enrich to sites of membrane curvature. Unique curvature preferences among WASP family NPFs could help to differentiate their organization and inform the morphology of the actin networks they generate. For N-WASP, enrichment to membrane invaginations could facilitate scission of endocytic vesicles from the plasma membrane (Kaksonen et al., 2005; Akamatsu et al., 2020). For WAVE, saddle preference could help to organize a coherently advancing flat lamellipod (Pipathsouk et al., 2021), and for WASP, enrichment to stable inward curvature (in particular the saddle-shaped necks of invaginations) could help the cell to integrate substrate topologies with motility. Interestingly, these proteins also appear to have different rules for reading curvature; for instance, WASP is much better than WAVE at persistently enriching to saddles at bead-induced membrane invaginations (Fig. 2, F and G). Whether these individualized responses arise from subtle differences in saddle curvature preference, WAVE complex’s extinction at nonmotile barriers (Weiner et al., 2007), or WASP’s broader permissive zone (Fig. 1 A) will be an interesting avenue for future investigation. Notably, in some contexts, WASP can even substitute for WAVE (Veltman et al., 2012; Zhu et al., 2016; Tang et al., 2013), suggesting an overlap in the membrane geometries they can read. Future studies systematically investigating how actin regulators sense and respond to membrane curvature will expand our understanding of the self-organizing rules that control cell morphogenesis.

## Materials and methods

### Cell culture

HL-60 cells are from the laboratory of Henry Bourne. PLB-985 cells were originally obtained from Onyx Pharmaceuticals. RNA sequencing was recently performed on both these backgrounds (Rincón et al., 2018), confirming cell line identity and supporting a previous report that PLB-985 cells are actually a subline of HL-60 cells (Drexler et al., 2003). Both lines were grown in RPMI 1640 media supplemented with L-glutamine and 25 mM Hepes (MT10041CV; Corning) and containing 10% (vol/vol) heat-inactivated fetal bovine serum (16140071; Gibco BRL) and 1× penicillin/streptomycin (15140148; Gibco BRL; called HL-60 media). Cultures were split to 0.17–0.2 million cells/ml every 2–3 d and grown at 37°C in 5% CO_2_. Cells were differentiated for experiments by diluting to 0.2 million/ml, adding 1.5% (vol/vol) DMSO (358801; Santa Cruz Biotechnology), and incubating for 4–5 d. HEK293T cells (used to generate lentivirus for transduction of HL-60 cells) were grown in DMEM (11995065; Gibco BRL) containing 10% (vol/vol) heat-inactivated fetal bovine serum and 1× penicillin/streptomycin and maintained at 37°C in 5% CO_2_. Cells were routinely checked for mycoplasma contamination.

### Plasmids

A vector for mammalian expression of TagRFP-T-Arp3 was generated by PCR amplification of the Arp3 coding sequence and Gibson assembly into a lentiviral pHR backbone containing an N-terminal TagRFP-T sequence. A similar approach was used to generate TagRFP-T-WASP-GBD. The design of this biosensor was based on the sequence used in Nalbant et al. (2004). The sequence for mNeonGreen2_1–10_ (mNG2_1–10_; Feng et al., 2017) was transferred to a pHR backbone. BFP-conjugated PAK-PBD was generated according to constructs used previously in Weiner et al. (2007) and Graziano et al. (2019). Hem1-eGFP and CAAX-tagBFP were previously described (Diz-Muñoz et al., 2016; Pipathsouk et al., 2021).

For generating lentivirus-based KO lines, gRNAs with homology to exon 1 of *WAS* (5′-CCA​ATG​GGA​GGA​AGG​CCC​GG-3′ and 5′-GCT​GAA​CCG​CTG​GTG​CTC​CT-3′) and exon 4 of *FNBP1* (5′-ACG​AAA​TGA​ATG​ATT​ACG​CA-3′) were selected using the CRISPR design tool in Benchling (https://www.benchling.com) and cloned into the previously described LentiGuide-Puro vector (Addgene plasmid #52963; Sanjana et al., 2014). The vector used to express human codon–optimized *Streptococcus pyrogenes* Cas9-BFP was also previously described (Graziano et al., 2017).

For endogenous tagging of WASP with plasmid donor (used in Fig. S2, B and C), 500 bp of the 5′ untranslated region and the start codon (5′ homology arm), TagRFP-T flanked by unique 7-amino acid-long linkers, and the 500 bp following the start codon (3′ homology arm) were Gibson assembled into a minimal backbone (pUC19). A similar approach was used for endogenous tagging of clathrin LCa (*CLTA*).

### Transduction of HL-60 cells

HEK293T cells (ATCC) were seeded into six-well plates and grown until ∼70% confluent. For each well, 1.5 μg pHR vector (containing the appropriate transgene), 0.167 μg vesicular stomatitis virus-G vector, and 1.2 μg cytomegalovirus 8.91 vector were mixed and prepared for transfection using TransIT-293 Transfection Reagent (MIR 2705; Mirus Bio). Following transfection, cells were incubated for 3 d, after which virus-containing supernatants were harvested and concentrated ∼40-fold using Lenti-X Concentrator (631232; Takara Bio) per the manufacturer’s instructions. Concentrated viruses were frozen and stored at −80°C until needed or used immediately. For all transductions, virus was mixed with 0.36 million cells in growth media supplemented with 8 μg/ml Polybrene (H9268; Sigma-Aldrich) and incubated overnight. FACS (FACSAria2 or FACSAria3; BD Biosciences) was used to isolate cells with the desired expression level of the transgene.

### Generation of CRISPR KO HL-60 cell lines

WASP and FBP17 HL-60 KO cell lines were generated as described in Graziano et al. (2019). Briefly, HL-60s were transduced with a puromycin-selectable vector containing an sgRNA sequence for the gene of interest. Following puromycin selection, cells were transduced with a *S*. *pyrogenes* Cas9 sequence fused to tagBFP. Cells expressing high levels of Cas9-tagBFP were isolated with FACS. Cells were then diluted into 96-well plates at a density of approximately one cell per well in 50% (vol/vol) filtered conditioned media from a healthy culture, 40% (vol/vol) fresh HL-60 media, and 10% (vol/vol) additional heat-inactivated fetal bovine serum. Clonal cell lines were expanded and validated using amplicon sequencing and immunoblot. Two clonal lines were generated for WASP from separate CRISPR assemblies (guide 1: 5′-CCA​ATG​GGA​GGA​AGG​CCC​GG-3′; guide 2: 5′-GCT​GAA​CCG​CTG​GTG​CTC​CT-3′). One CRISPR assembly was used for FBP17 (5′-ACG​AAA​TGA​ATG​ATT​ACG​CA-3′), and clonal lines were isolated and validated with both sequencing and immunoblot. A single clone was then selected and further edited to remove CIP4.

Subsequent KO of CIP4 in FBP17 KO cells was done through electroporation with a CRISPR-Cas9 RNP complex as in Brunetti et al. (2018). Details of this process are outlined in the section Generation of CRISPR KI HL-60 cell lines. The only difference between these protocols is inclusion or exclusion of a repair template (exclusion in the case of KO). The following guides were selected using the CRISPR design tool in Benchling (https://www.benchling.com): 5′-cgg​cgg​GAG​CAG​CAT​GGA​TT-3′ or 5′-GCT​CGA​GCG​CCA​CAC​GCA​GT-3′. Edited clones were successfully isolated for both guides and validated by sequencing.

The Cdc42 PLB-895 KO line was generated as described in Bell et al., (2021; *Preprint*). Briefly, PLB-895 cells were electroporated with a CRISPR-Cas9 RNP complex. Tandem guides targeting exon 4 of Cdc42 were purchased from Synthego (5′-TTT​CTT​TTT​TCT​AGG​GCA​AG-3′ and 5′-ATT​TGA​AAA​CGT​GAA​AGA​AA-3′). Guides were complexed with Cas9 protein and electroporated into cells using a custom suspended-drop electroporation device (Guignet and Meyer, 2008).

### Immunoblot assays

Protein content from 1 million cells was extracted using TCA precipitation. Samples were separated via SDS-PAGE and transferred to a nitrocellulose membrane. Membranes were blocked for ∼1 h in a 1:1 solution of TBS (20 mM Tris, 500 mM NaCl [pH 7.4]) and Odyssey Blocking Buffer (927-70001; LI-COR). For detection of WASP, the membrane was incubated overnight at 4°C in primary antibody (rabbit, 4860; Cell Signaling Technology) diluted 1:1,000 in a 1:1 solution of TBS + 0.2% wt/vol Tween 20 (TBST) and Odyssey Blocking Buffer. GAPDH (mouse, MA5-15738; Invitrogen) was used as a loading control. Membranes were washed three times with TBST and then incubated for 45 min at room temperature with LI-COR secondary antibodies (goat anti-rabbit and goat anti-mouse; LI-COR 926–32211 and 926–68070) diluted 1:20,000 in either Odyssey Blocking Buffer. Membranes were then washed three times with TBST, washed a final time with TBS, and imaged using an Odyssey Fc Imaging System (LI-COR).

### Generation of CRISPR KI HL-60 cell lines

WT HL-60 cells were transduced with a plasmid containing the nonfluorescent 1–10 segment of mNG2_1–10_ (Feng et al., 2017). Cells expressing the transgene were identified through electroporation (4D-Nucleofector; Lonza) with an mCherry-conjugated mNG2_11_ mRNA. Positive cells (those that were fluorescent at both 488 nm and 561 nm excitation) were identified using FACS (SH800; Sony), and a subset of cells that appeared to have a single insertion of mNG2_1–10_ was isolated. After a few days, the mRNA was degraded, and the cells lost fluorescence. This created the base mNG2_1–10_ HL-60 cell line used with mNG2_11_ KI.

sgRNAs of the target gene were obtained by in vitro transcription as described in Leonetti et al. (2016) with modifications only to the PCR polymerase used (Phusion, M0530S; New England Biolabs). A 100-μl reaction of mNG2_1–10_ HL-60 cells were electroporated (Neon; Invitrogen) with a Cas9–sgRNA complex and a single-stranded DNA ultramer (ssDNA; Integrated DNA Technologies) donor containing mNG2_11_, a linker, and ∼55 bp of homology to each side of the cut site. Cas9–sgRNA complex formation and electroporation protocols were adapted from Brunetti et al., (2018) and Garner et al. (2020). Briefly, 90 pmol of Cas9 containing a nuclear localization signal (QB3 MacroLab) and 270 pmol of sgRNA were mixed on ice and then incubated at room temperature for 30 min. Meanwhile, 2 million cells were spun down at 300 x*g* for 5 min and washed with PBS to remove extracellular proteins. When the Cas9 complex was ready, cells were again spun down and this time resuspended in 100 μl room temperature R buffer (MPK10096; Invitrogen). 100 pmol of ssDNA donor was added to the Cas9–sgRNA complex, and the whole mixture was added to the resuspended cells. Samples were then electroporated at 1,350 V for 35 ms and recovered in 5 ml warmed media. Cells were monitored and allowed to recover. Using 100-μl tips led to recovery within only a few days. Cells were then expanded for selection with FACS. The percentage of fluorescent cells ranged from 0.1 to 1%. For *WAS*, which has only one allele, the polyclonal line resulting from FACS sorting was sequence verified and used for experiments. For other KIs, cells underwent clonal dilution as outlined above, and surviving clones were screened using a PCR-based gel shift assay. Samples showing biallelic insertion were gel extracted and Sanger sequenced.

In the case of full-length fluorophore KI, plasmid donor was used in place of ssDNA donor. The above protocol was executed with only slight changes. First, 10-μl tips were used in place of 100-μl tips to remove the need for large amounts of donor. RNP components and the number of cells electroporated were therefore scaled down by a factor of 10 as originally done in Brunetti et al. (2018) and Garner et al. (2020). Next, 800 ng of plasmid donor was provided in place of the ssDNA donor. Finally, after electroporation, cells were rescued in 500 μl prewarmed media in a 24-well plate and allowed to recover for 10–14 d. Despite lowered viability and longer recovery times after electroporation compared with 100-μl tips, similar tagging efficiencies were observed with this strategy.

N-terminal tagging was performed for WASP (*WAS*), clathrin LCa (*CLTA*), and FBP17 (*FNBP1*). We used the following guide sequences to target near the start codon: *WAS*, 5′-GGC​AGA​AAG​CAC​CAT​GAG​TG-3′; *CLTA*, 5′-GAA​CGG​ATC​CAG​CTC​AGC​CA-3′; and *FNBP1*, 5′-CGT​CCC​CTG​CAC​CAT​GAG​CT-3′.

### Under-agarose preparation of HL-60s for imaging

For all experiments except the STED, collagen, nanopatterns, and EDTA treatment, HL-60s were prepared via a standard under-agarose protocol (Bell et al., 2018). For this work, a solution of 2% low-melt agarose (A-204; Gold Biotechnology) was made in L-15 media (21083-027; Gibco BRL) and microwaved in a loosely capped conical tube placed in a water reservoir. Heating was done in short increments to promote melting while preventing the solution from boiling over. Once completely melted, the gel was kept at 37°C to allow cooling while preventing solidification. Meanwhile, 500 μl of differentiated HL-60 cells were spun down at 200 x*g* for 5 min and then concentrated threefold in HL-60 media. 5 μl of the concentrated cells were placed in the center of a circular well (655891; Greiner Bio-One) and allowed to settle. When circular-welled plates could not be used, a 5-mm circular mold was inserted into the well. After the agarose had cooled to 37°C, 195 μl of agarose was slowly pipetted into the bottom edge of the well, allowing the agarose to spread over the droplet of cells. The pipette was then slowly raised up the side of the well to continue depositing agarose without sweeping away the covered cells. The plate was then allowed to incubate for 20–45 min at 37°C.

An agarose preparation was also used in STED experiments, but modifications were used to accommodate imaging chambers (80827; Ibidi). Briefly, a 1% solution of liquid agarose (17850; Thermo Fisher Scientific) was made and poured into 8 × 8 × 5–mm square molds. After solidification, agarose pads were stored in L-15 media at 4°C. Prior to imaging, a room temperature agarose pad was placed atop the sample with tweezers.

### Thin slide preparation of HL-60s for imaging

For EDTA experiments, thin slide preparations were used to maintain the polarity and migration of cells in the absence of integrin-based adhesions. To achieve this, cells were plated between two glass surfaces as described in Malawista et al. (2000) and Graziano et al. (2017). Briefly, glass slides and coverslips were thoroughly cleaned with Fisherbrand Sparkleen laboratory detergent (04-320-4; Fisher Scientific) and then sonicated for 10 min first in acetone, then twice in ethanol, and finally three times in MilliQ water. The glass was immediately dried using N_2_ gas and stored in a clean, dry place. For experiments, differentiated HL-60s were concentrated to 20 million cells/ml in HL-60 media, and a final concentration of 10 mM EDTA (E0306; Teknova) was added to “+EDTA” samples. 2 μl of cells were then placed onto a clean slide and a clean 18 × 18–mm coverslip was dropped on top. We confirmed that the cell solution was wicked across the majority of the coverslip surface. Chambers were then sealed with VALAP (a mixture of equal parts Vaseline, paraffin wax, and lanolin; Cold Spring Harbor Laboratory, 2015) and imaged.

### Cell migration on beads

An under-agarose preparation was performed as described above with the addition of red fluorescent (580/605) carboxylate-modified microspheres (F8887; Invitrogen) to concentrated cells before plating. For quantitation of WASP and Arp2/3 recruitment to beads, a low density of beads was used (1:10,000 to 1:100,000). For bead carpets, as shown in Fig. 4 A, beads were diluted only 1:1,000. Since bead density is proportional to the bead volume, a less stringent dilution was used for 500-nm beads (1:100).

### STED microscopy of cells on beads

All STED microscopy experiments were performed in plain glass eight-well chambers (80827; Ibidi). Wells were coated with 100 μl of 20 μg/ml porcine fibronectin (prepared from whole blood) diluted in PBS at 37°C for 30 min and washed three times using L-15 media (21083-027; Gibco BRL). Next, any residual imaging media was removed, and 100 μl of beads diluted in L-15 were added to the well. We used red fluorescent (580/605) carboxylate-modified beads of 200 or 500 nm diameter (F8887; Invitrogen) and nonfluorescent carboxylate-modified beads of 500 nm diameter (C37481; Invitrogen). Bead density and adhesion were checked on the microscope before the addition of cells. Next, 800 μl of differentiated HL-60 cells were spun down at 200×*g* for 5 min and resuspended in 100 μl of 5× concentrated CellMask Deep Red (C10046; Invitrogen) in L-15. Immediately after resuspension in the labeling solution, cells were spun down again at 200×*g* for 5 min. After complete removal of supernatant, cells were resuspended in 100 μl of L-15 media and added to the well containing beads. To promote cell adhesion, the sample was placed in a 37°C incubator for 15 min. Finally, an agarose pad was placed atop cells, and samples were imaged at room temperature.

### Cell migration on fibronectin-coated coverslips

The desired number of wells in a 96-well plate (MGB096-1-2-LG-L; Matrical, Inc.) were coated with 100 μl of 20 μg/ml porcine fibronectin (prepared from whole blood) dissolved in PBS for 30 min at room temperature. The fibronectin solution was then removed and replaced with 100 μl of undiluted differentiated HL-60s. The imaging plate was then transferred to a 37°C/5% CO_2_ incubator for 15 min to allow cells to adhere. Wells were then gently washed with HL-60 media, and finally the solution was exchanged for 100 μl of fresh HL-60 media. The plate was next transferred to the microscope, which had been preheated to 37°C, for imaging.

### Cell migration on nanopatterned substrates

Nanopatterned and flat control substrates were purchased in 96-well plate format from Curi Bio (ANFS-0096). Wells were coated with 50 μl of 10 μg/ml porcine fibronectin (prepared from whole blood) dissolved in PBS for 1 h at room temperature. The fibronectin solution was then removed and replaced with differentiated HL-60s (WT or WASP KO) diluted by a factor of five into fresh HL-60 media containing one drop of the nuclear dye NucBlue (R37605; Thermo Fisher Scientific) per 500 μl. The plate was then transferred to a 37°C/5% CO_2_ incubator for 15 min to allow cells to adhere. The medium was then gently pipetted over cells a few times to remove unattached cells and the media was exchanged for 100 μl fresh HL-60 media without NucBlue. The plate was then transferred to the microscope, which had been preheated to 37°C, and an environmental chamber supplying 5% CO_2_ was turned on. The plate was allowed to equilibrate for 1 h before imaging, giving cells time to align on nanopatterns. WT and WASP KO cells were always plated in adjacent wells so that three positions could be taken per well over the time interval between frames (45 s). Acquisition lasted 1 h, and both the transmitted light and 405 nm (nuclei) channels were collected.

### Cell migration in fluorescent collagen matrices

30 μl of pH-adjusted FITC-conjugated bovine skin collagen at 1 mg/ml (C4361; Sigma-Aldrich) was polymerized in a round, glass-bottomed well (655891; Greiner Bio-One) for 3 min at room temperature and then gently washed with PBS (Elkhatib et al., 2017). 100 μl of differentiated HL-60s in HL-60 media was immediately added and allowed to migrate into the network up to 1 h before imaging. WASP colocalization with fibers was assessed using a cell line that had WASP endogenously tagged with TagRFP-T.

### Microscopy

TIRF imaging experiments in Fig. 1 (except panel E), Fig. 2, and Fig. 4 (except panel F) were performed at room temperature using the ring TIRF light path of a DeltaVision OMX SR microscope (GE Healthcare) with a 60×/1.42 NA oil Plan Apochromat objective (Olympus) and 1.518 refractive index oil (Cargille). The system is equipped with 405-, 445-, 488-, 514-, 568-, and 642-nm laser lines. TagBFP-tagged CAAX was imaged with the 405-nm laser line, mNG2 and eGFP-tagged proteins were imaged with the 488-nm laser line, and TagRFP-T–tagged proteins were measured with the 568-nm laser line. The system was controlled with OMX AquireSR software (GE Healthcare), and alignment of two channel images was performed with SoftWoRx (GE Healthcare).

TIRF and confocal imaging experiments in Fig. 1 E, Fig. 4 F, Fig. 5, Fig. 6 (except panel G), and Fig. 7 were performed at 37°C on a Nikon Eclipse Ti inverted microscope equipped with a motorized laser TIRF illumination unit, a Borealis beam conditioning unit (Andor), a CSU-W1 Yokugawa spinning disk (Andor), 60× and 100× Plan Apochromat TIRF 1.49 NA objectives (Nikon), an iXon Ultra EMCCD camera (Andor), and a laser module (Vortran Laser Technology) equipped with 405-, 488-, 561-, and 642-nm laser lines. NucBlue was measured with the 405-nm line, mNG2-tagged proteins were measured with the 488-nm line, and TagRFP-T–tagged proteins were measured with the 561-nm line. The system was controlled with μManager software version 2.0-β (Edelstein et al., 2010).

Confocal microscopy and STED microscopy for Fig. 3 and Fig. 6 G were performed at room temperature using an Abberior Expert Line system (Abberior Instruments GmbH) with an inverted Olympus IX83 microscope, QUADScan Beam Scanner (Abberior Instruments GmbH), and an Olympus UPlanSAPO 100×/1.41 NA oil immersion objective lens. The system was controlled with ImSpector (Abberior). Confocal measurements of mNG2 were made using a 488-nm line. All STED measurements were made using a 640-nm line and a 775-nm STED depletion laser. Sequential imaging was used to avoid cross excitation. 2D STED imaging of the CellMask Deep Red and SiR-actin were performed using a 30-nm pixel size for x and y. 3D STED imaging of CellMask Deep Red was performed in xzy mode using a z piezo stage (P-736 PInano; Physik Instrumente), the Abberior adaptive illumination module RESCue, and a 30-nm voxel size for x, y, and z (Staudt et al., 2011). The STED and confocal channels were aligned using measurements of 100-nm fluorescent beads on the Abberior autoalignment sample. Spatial resolution of 3D STED was determined by imaging 40-nm far-red fluorescent beads purchased from Abberior.

### Image analysis

Images were displayed in Fiji, and WASP puncta and nuclei tracking were performed using the plug-in TrackMate (Tinevez et al., 2017). Tracks were filtered for desired property (duration, quality, etc.) within TrackMate, and coordinates were exported as CSV files and analyzed with custom Python code that made heavy use of the scikit-image package (van der Walt et al., 2014). All analysis code and a CSV of all data are available at https://github.com/rachel-bot/WASP. Kymographs were created using the plug-in KymographBuilder (Mary et al., 2016).

### Spatial histogram analysis

Using cells oriented from left to right, a cell mask and its contour were created. The left, right, top, and bottom bounds of the cell outline were extracted. The width of the cell in x was divided into the specified number of bins, here 10. Next, a difference of Gaussians image of WASP or clathrin or Arp3 was generated, and a weighted Otsu threshold was applied. The user confirmed that this scheme of puncta identification was sufficiently robust. The resulting binary image was indexed, and puncta centroids were recorded. The x coordinate of each puncta was then used to assign it to one of the bins made from dividing the cell mask’s x coordinate range into the specified number of bins. A similar analysis was repeated over all cells. Puncta counts per bin (each with different x coordinates but representing the same portion of the cell, i.e., the front 1/10th) from all cells were then combined and normalized to the total puncta counts to get a density. For display purposes, this 1D array was extended in y to be 2D and multiplied by the binary mask of a well-polarized cell. All values outside the cell body were converted to NaN (not a number) to give a white background, and the cell contour was plotted on top to denote this boundary.

Similar analysis was employed to investigate the disappearance of WASP puncta in the ±EDTA experiments. To do this, puncta tracks were determined in Fiji using TrackMate (Tinevez et al., 2017), discarding traces that did not disappear during the recorded timelapse. All tracks were manually checked to ensure that they accurately captured the last frame of the puncta. Centroids at these times points were then passed to the analysis described above, and their position relative to the cell length was determined by manual specification of the cell front and cell back in each frame using the Matplotlib (Hunter, 2007) command “ginput” to select these physical landmarks.

### Characterizing puncta dynamics in the cell frame of reference

Cells were oriented so that their migration was roughly left to right. A binary of the cell image was created for each frame, and the center of mass of each was aligned. This centered mask was then populated with the corresponding WASP intensity values from the original timelapse. Puncta trajectories were generated with the Fiji plug-in TrackMate (Tinevez et al., 2017). Because of high puncta density and subsequent trajectory mismapping, each track was manually checked for accuracy. This led to a subsampling of puncta across cells. For display purposes, cell trajectories were overlaid on the microscopy data as a function of time and were color-coded by temporal position relative to the puncta’s whole duration. This analysis was repeated over many cells, and the first and last values of each trajectory were extracted for comparison of puncta appearance and disappearance.

To determine relative position of appearance and disappearance, the cell front (center) and rear were selected for each frame containing an appearing or disappearing puncta. The line from the cell front to the puncta was then projected onto the line running from the cell front to rear. To calculate the fraction of this value relative to cell length, we then divided this value by the length of the line running from cell front to rear. This scheme was repeated for the y dimension by selecting the cell’s top and bottom. This allowed 2D position comparisons across frames and across cells.

### Puncta identification and quantification

To compare WASP puncta formation across genetic backgrounds, local maxima were detected in a difference of Gaussians image of flatfield-corrected WASP taken from the middle of a timelapse of a persistently migrating cell. A threshold was determined empirically that was generous in what it deemed a local maxima so as to not discount potentially dimmer puncta in the mutant (Cdc42-null) background. Because of the lax gating, the user had to manually confirm whether a local maximum was in fact a puncta in the original image, as opposed to noise or a cell edge. The number of puncta per cell was tallied. For intensity values, each puncta was fit with a 2D Gaussian, and the background-independent signal was calculated using the equation for volume under a Gaussian 2πAσ_x_σ_y_, where the amplitude (A) and the standard deviation in each direction (σ_x_, σ_y_) come from the fit. Finally, the local areas surrounding puncta centroids were projected in time to span the entire timelapse, and the user selected the appearance and disappearance frames for each puncta to get puncta lifetime. Puncta appearing before the timelapse start or persisting past the timelapse end were omitted from this calculation.

### Quantification of WASP and Arp3 signal at beads

Single frames from timelapses of cells migrating over beads were selected based on the presence of beads under the front half of the cell. The Matplotlib (Hunter, 2007) command “ginput” was used to manually select beads that were completely under the cell. The immediate region around the bead was isolated, and a 2D Gaussian was fit to the WASP or Arp3 signal. Intensity of the signal at the bead was calculated using the formula for volume under a Gaussian 2πAσ_x_σ_y_, where the amplitude (A) and the standard deviation in each direction (σ_x_, σ_y_) come from the fit. This quantity is independent of background signal. For comparing WASP intensity across bead sizes, Gaussian volumes were normalized to bead surface area in accordance with our observation from STED microscopy that WASP signal is distributed across the entire invagination surface at sufficiently high curvatures (≤1/200 nm). For assessing Arp3 recruitment to beads in WT and WASP KO cells, Gaussian volumes were not directly compared, since many beads failed to induce Arp3 puncta in the absence of WASP and, therefore, could not be fit. Instead, we reported the fraction of beads able to form Arp3 puncta for each cell background. To avoid losing the intensity data entirely, we took an orthogonal approach and calculated a background-subtracted integrated intensity for Arp3 signal at beads. To do this, Arp3 signal was tracked over time, and the frame with the brightest Arp3 signal in the region of the bead of interest was isolated. Arp3 signal was then masked with the bead signal to separate out the foreground and background. The mean background signal was calculated and subtracted from each pixel in the masked image. The resulting values were then summed to get the background-subtracted Arp3 integrated intensity.

### Measuring WASP signal as a function of distance from the leading edge

Timelapses where a cell moved the majority of its length over a bead without dislodging it from the TIRF plane were analyzed. At each time point, the cell rear and cell front were manually selected with the Matplotlib (Hunter, 2007) command “ginput” to find the cell’s major axis. Beads that passed under the cell were selected, and at each time point, their (stationary) position was projected onto the major axis. This value was normalized to the major axis length in each frame to get a relative distance from the cell front (value of 0–1). For each time point, WASP intensity was estimated by summing the signal in a small box around the bead. The exactness of this measurement is not imperative, since all traces are normalized to their maximum integrated WASP signal to build a profile of relative signal as a function of distance from the cell front.

To get a population-level profile, relative distances were discretized to the nearest 0.1, and traces from many beads were averaged to create a line plot. To better represent the data underlying this line, WASP data points in each positional bin were overlaid onto the plot and colored by the kernel density estimate of the value compared with all WASP values in the same positional bin.

### Colocalization by correlation analysis

Colocalization of curvature sensors with beads and WASP with clathrin or Arp3 or FBP17 was determined by calculating the Pearson correlation coefficient between the flattened vectors of each channel. For statistical comparison of enrichment between curvature sensors, distributions of correlation coefficients were compared using an unpaired two-tailed *t* test. For statistical comparison of colocalization between markers in the same cell, a negative control was generated by rotating one channel 90° to remove colocalization while maintaining any punctate organization in the image (Dunn et al., 2011). The Pearson correlation coefficient was again calculated. The values of nontransformed and transformed correlation coefficients were combined across cells and experiments to create distributions. The means from each technical replicate were then compared between these two conditions using a paired *t* test.

### Cell tracking and measurement of migration properties

Cell nuclei were tracked in Fiji using TrackMate (Tinevez et al., 2017), and resulting CSV files were exported for analysis in Python.

To prevent double counting of cells, only traces that lasted at least half of the timelapse were kept. Then, to make sure all cells were compared over the same time window, only cells present for the first half of the timelapse were analyzed. Total distance traveled by each cell was calculated by applying the distance formula to each time point and summing the resulting displacements. This was performed for either both x and y or x or y independently, depending on the kind of distance being reported.

MSD was calculated using

$$\frac{1}{N}\sum_{i=1}^{N}{\left|x^{\left(i\right)}(t)-x^{\left(i\right)}(0)\right|}^{2},$$

where $x^{\left(i\right)}(t)$ is the position of i*-*th cell at time *t* and $x^{\left(i\right)}(0)$ is its initial position. *N* denotes the total number of cells.

The persistence ratio for each cell was calculated using

$$\frac{\left|x(T)-x(0)\right|}{\sum_{t=0}^{T}\left|x(t)-x(0)\right|},$$

where T denotes the time point being compared with *t* = 0 (Gorelik and Gautreau, 2014). The persistence ratio traces were then averaged across cells.

### Statistics and reproducibility

All experiments were repeated a minimum of three times unless otherwise specified. For comparisons between datasets in Fig. 2 E, Fig. 5, Fig. 6, and Fig. 7, the mean of each technical replicate is plotted as a large colored marker over the single-cell distribution. When there were many observations per cell, statistics were done on single-cell means. Otherwise, statistics were done on replicate means. Alternatively, in the case of small *n*, such as in Fig. 2 G and Fig. 3 D, statistics were applied to all measurements. We used a paired *t* test when datasets were related (i.e., localization of clathrin and WASP in the same cells (Fig. 1 D) or WASP signal at single beads before and after membrane closing (Fig. 3 D) or if there was obvious technical variability (Fig. 5 F). We also used paired *t* tests to compare migration properties as suggested in Lord et al., (2020) to reduce influence from day-to-day variability. For measurements that do not exhibit major experimental variation (such as the position of WASP puncta disappearance; Fig. 1 F), we used an unpaired *t* test. Paired and unpaired two-tailed *t* tests are standard for comparing means between parametric datasets. Normality was assumed under the central limit theorem but was not formally tested.

### Online supplemental material

Fig. S1 shows a schematic of the KI strategy used and reports the propensity of HL-60 cells to undergo nonhomologous end joining and homology-directed repair; it also includes sequencing validation of the endogenously tagged WASP and clathrin LCa cell lines, as well as Western blot and sequencing validation of WASP KO cell lines. Fig. S2 shows that endogenous WASP puncta form in the absence of agarose overlay and that puncta enrich to the interface between HL-60 cells and collagen fibers. Fig. S3 examines the relationship between WASP and FBP17/CIP4. Fig. S4 demonstrates the resolution increase provided by 3D STED and offers an orthogonal measure of WASP enrichment as a function of bead diameter. Fig. S5 further investigates how polarity informs WASP recruitment. Video 1 shows the dynamics of endogenous WASP in a persistently migrating HL-60 cell. Video 2 shows the spatial separation of endogenous WASP and endogenous clathrin LCa in an HL-60 cell. Video 3 shows the effect of blocking integrin-based adhesion on WASP puncta dynamics. Video 4 shows the dynamics of WASP recruitment to sites of bead-induced plasma membrane deformation. Video 5 shows plasma membrane and WASP rearrangement as an HL-60 cell migrates over beads. Video 6 shows WASP puncta dynamics in the frame of reference of a cell. Video 7 shows similar migration between WT and WASP KO HL-60 cells on flat substrates. Video 8 shows significant defects in WASP KO HL-60 cells plated on nanoridged substrates.

## Data Availability

All analysis code and a CSV of all data are available at https://github.com/rachel-bot/WASP.
